# Unlocking Intracellular Protein Delivery by Harnessing Polymersomes Synthesized at Microliter Volumes using Photo‐PISA

**DOI:** 10.1002/adma.202408000

**Published:** 2024-10-17

**Authors:** Chalaisorn Thanapongpibul, Omar Rifaie‐Graham, Miina Ojansivu, Adrian Najer, Hyemin Kim, Saskia E. Bakker, Mohamed Chami, David J. Peeler, Chenchen Liu, Jonathan Yeow, Molly M. Stevens

**Affiliations:** ^1^ Department of Materials, Department of Bioengineering, and Institute of Biomedical Engineering Imperial College London London SW7 2AZ UK; ^2^ Department of Medical Biochemistry and Biophysics Karolinska Institutet Stockholm 17177 Sweden; ^3^ Advanced Bioimaging Research Technology Platform University of Warwick Gibbet Hill Road Coventry CV4 7AL UK; ^4^ BioEM Lab Biozentrum University of Basel Basel 4058 Switzerland; ^5^ Kavli Institute for Nanoscience Discovery Department of Physiology, Anatomy and Genetics Department of Engineering Science University of Oxford Oxford OX1 3QU UK; ^6^ Graduate School of Biomedical Engineering University of New South Wales Sydney NSW 2052 Australia

**Keywords:** intracellular delivery, pH responsive, polymerization‐induced self‐assembly, polymersomes, protein delivery

## Abstract

Efficient delivery of therapeutic proteins and vaccine antigens to intracellular targets is challenging due to generally poor cell membrane permeation and endolysosomal entrapment causing degradation. Herein, these challenges are addressed by developing an oxygen‐tolerant photoinitiated polymerization‐induced self‐assembly (Photo‐PISA) process, allowing for the microliter‐scale (10 µL) synthesis of protein‐loaded polymersomes directly in 1536‐well plates. High‐resolution techniques capable of analysis at a single particle level are employed to analyze protein encapsulation and release mechanisms. Using confocal microscopy and super‐resolution stochastic optical reconstruction microscopy (STORM) imaging, their ability to deliver proteins into the cytosol following endosomal escape is subsequently visualized. Lastly, the adaptability of these polymersomes is exploited to encapsulate and deliver a prototype vaccine antigen, demonstrating its ability to activate antigen‐presenting cells and support antigen cross‐presentation for applications in subunit vaccines and cancer immunotherapy. This combination of ultralow volume synthesis and efficient intracellular delivery holds significant promise for unlocking the high throughput screening of a broad range of otherwise cost‐prohibitive or early‐stage therapeutic protein and vaccine antigen candidates that can be difficult to obtain in large quantities. The versatility of this platform for rapid screening of intracellular protein delivery can result in significant advancements across the fields of nanomedicine and biomedical engineering.

## Graphical abstract

Efficient intracellular delivery of therapeutic proteins is challenging due to poor cell membrane permeation and endolysosomal entrapment. An oxygen tolerant Photo‐PISA process is reported for the microliter‐scale synthesis of polymersomes that deliver proteins into the cytosol and activate antigen‐presenting cells. This high throughput approach is a highly promising strategy for the screening of early‐stage therapeutic proteins and vaccine antigen candidates.

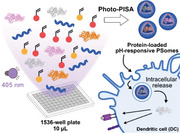

## 1 Introduction

Proteins have attracted increasing attention for biomedical applications in recent years owing to their significant potential in precisely modulating many key biochemical processes such as cellular metabolism, signaling pathways, and molecular transport.^[^ ^1^ ^]^ This diversity has seen their application across several areas such as cancer therapy, vaccinology, immunotherapy, and non‐gene‐based approaches to enzyme replacement therapy.^[^ ^1^, ^2^ ^]^ Biomacromolecules as therapeutics offer distinct advantages compared to conventional small molecule drugs, particularly with regards to their highly evolved specificity which can be attributed to their complex yet well‐defined macromolecular structure and bio‐physical interactions.^[^ ^3^ ^]^ However, the efficient intracellular delivery of therapeutic proteins remains a significant challenge due to the broad range of inhibitory components and other barriers such as susceptibility to proteolytic degradation, poor permeation through cell membranes, and endosomal entrapment and degradation.^[^ ^4^ ^]^ To overcome these limitations, protein delivery vehicles have been developed that provide physicochemical protection from degrading agents, improve cellular uptake, and aid in endosomal escape, thereby enabling controlled release and targeted delivery.^[^ ^5^ ^]^ It should be noted, however, that versatile intracellular delivery platforms that meet this need, while also being highly conducive for in vitro screening applications, are rare.

Polymeric vesicles, also known as polymersomes (PSomes),^[^ ^6^ ^]^ composed of assemblies of amphiphilic block copolymers are particularly promising as nanocarriers for intracellular protein delivery owing to their ability to compartmentalize and provide stabilization for proteins in their aqueous lumen.^[^ ^7^ ^]^ Compared to liposomes, the chemical diversity and tuneability of PSomes can allow for greater control over their physicochemical stability as well as stimuli‐responsive behaviors, enabling highly precise and controlled drug delivery.^[^ ^8^ ^]^ Conventional PSome self‐assembly methods involve step‐wise polymer synthesis, purification, self‐assembly, and purification. In contrast, polymerization‐induced self‐assembly (PISA) is a particularly attractive one‐step chemical self‐assembly method. In this process, a hydrophilic polymer is chain‐extended with a hydrophobic monomer via controlled/living polymerization, which drives in situ self‐assembly of the growing amphiphilic chains.^[^ ^9^ ^]^

Recently, researchers developed photoinitiated PISA (Photo‐PISA),^[^ ^10^ ^]^ as a facile method for in situ protein compartmentalization, coupling polymer synthesis, self‐assembly, and protein encapsulation in a single‐step process for applications such as nanoreactors for biocatalysis^[^ ^11^ ^]^ or artificial organelles (AOs).^[^ ^12^ ^]^ Additionally, oxygen tolerant^[^ ^13^ ^]^ polymerization strategies were integrated to enable Photo‐PISA to be performed at ultralow microliter scales (10 µL) enabling the down‐scaled and cost‐efficient production of protein‐loaded PSomes without the need for specialized microfluidic approaches.^[^ ^12^, ^14^ ^]^ This approach overcomes limitations of conventional physical self‐assembly techniques such as thin film rehydration which are typically performed at milliliter scales and therefore require substantial quantities of proteins that might be expensive or otherwise difficult to acquire in sufficient quantities. By reducing the production scale of PSome syntheses, this approach is therefore highly conducive toward pilot experiments as well as high throughput combinatorial screening of potential intracellular therapeutics.

It should be noted, however, that while the high thermodynamic stability of PSomes compared to their low molecular weight liposome counterparts makes them highly suitable for application as catalytic nanoreactors (when permeable to small molecules),^[^ ^15^ ^]^ they generally require significant chemical modification to allow for controlled PSome disassembly and subsequent release of encapsulated macromolecules such as proteins.^[^ ^16^ ^]^ To this end, various stimuli‐responsive PSome systems have been proposed for the delivery of therapeutic proteins and vaccine antigens.^[^ ^17^ ^]^ However, the development of high throughput assembly techniques at ultralow scales, which allow the use of much smaller amounts of protein for initial testing, remains to be developed for protein delivery applications.

The entrapment of molecules within endosomes presents a notable obstacle to achieving effective intracellular delivery.^[^ ^18^ ^]^ A promising strategy for inducing the release of macromolecules intracellularly is by targeting the endolysosomal environment. During endocytosis, nanoparticles transition from the extracellular environment (pH = 7.4) to acidic microenvironments in early endosomes (pH = 5.9 – 6.8) which gradually mature into late endosomes and fuse with lysosomes (pH = 4.9 – 6.0).^[^ ^19^ ^]^ By exploiting the chemical tuneability of polymersome systems to present acid protonatable tertiary amine moieties,^[^ ^20^ ^]^ this acidic microenvironment can be exploited to enable endosomal escape of encapsulated cargo into the cytosol via proton sponge‐like effects.^[^ ^21^ ^]^ However, the exact mechanism and dynamics of these processes are still generally understood poorly and likely vary significantly between systems.^[^ ^18^, ^22^ ^]^ In recent studies, researchers have increasingly turned to the application of image‐based analytical methodologies, such as stimulated emission depletion microscopy (STED)^[^ ^23^ ^]^ and stochastic optical reconstruction microscopy (STORM),^[^ ^24^ ^]^ to investigate the underlying mechanisms behind endocytosis and endosomal escape.

**Figure 1:**
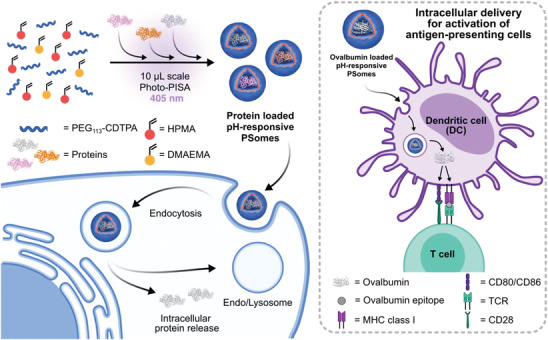
Microliter scale Photo‐PISA process enables the loading of various proteins into pH‐responsive PSomes to facilitate intracellular delivery and endosomal escape. This process is highly amenable to high throughput screening applications and in this study is used to demonstrate the delivery of ovalbumin as a model vaccine antigen to promote the maturation and activation of bone‐marrow derived dendritic cells.

Herein, we report a versatile, ultralow volume synthetic platform for producing pH‐responsive protein‐loaded PSomes that can efficiently deliver functional proteins into the cytoplasm (**Figure** **1**). Two distinct high resolution analytical techniques: nanoparticle flow cytometry (NanoFCM) and fluorescence correlation spectroscopy (FCS) are applied to study the loading and distribution of encapsulated proteins during PISA. Using labeled cargo, we also demonstrate the ability of these PSomes to release their encapsulated protein cargo under conditions mimicking the endolysosomal environment. To further investigate this intracellular release mechanism and to observe the endosomal escape process within cells, we apply conventional confocal microscopy as well as STORM as a super‐resolution analytical technique to visualize the localization of protein cargo after cellular uptake. As a proof‐of‐concept, we demonstrate the effectiveness of this system in delivering functional proteins to the cytosol. We achieved this by utilizing our PSome constructs to facilitate the cross‐presentation of ovalbumin (OVA) as a model vaccine antigen in bone marrow‐derived dendritic cells (BMDCs). Within this model, we illustrate that disassembly of pH‐responsive PSomes is essential for activating BMDCs and facilitating cross‐presentation of processed OVA antigen, leading to specific immune activation. This highlights the importance of dynamic nanoparticle design in navigating intracellular barriers to delivery. Together, this work demonstrates the development of a versatile low‐volume synthetic platform for protein‐loaded PSomes that can facilitate the in vitro screening of intracellularly‐active proteins for a broad range of potential applications in the biomedical space, such as vaccination and cancer immunotherapy.

## 2 Results and Discussion

### 2.1 Fabrication of pH‐Responsive PSomes at Microliter Volumes

**Figure 2:**
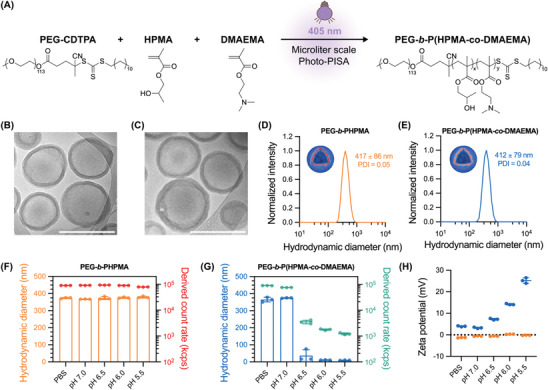
Synthesis and characterization of PSomes produced using a microliter scale Photo‐PISA platform. A) Chemical scheme showing the synthesis of PEG‐*b*‐P(HPMA‐*co*‐DMAEMA) via Photo‐PISA at a 10 µL scale. Representative cryo‐TEM images of B) PEG‐*b*‐PHPMA and C) PEG‐*b*‐P(HPMA‐*co*‐DMAEMA) PSomes. Scale bars: 500 nm. Average DLS intensity‐based distribution of D) PEG‐*b*‐PHPMA and E) PEG‐*b*‐P(HPMA‐*co*‐DMAEMA) PSomes. Variations in number‐based hydrodynamic diameter (bar) and derived count rate (scatter) as a function of pH for F) PEG‐*b*‐PHPMA and G) PEG‐*b*‐P(HPMA‐*co*‐DMAEMA) PSomes (*n* = 3, technical replicates). H) Zeta potential averages as a function of pH for PEG‐*b*‐PHPMA (orange) and PEG‐*b*‐P(HPMA‐*co*‐DMAEMA) PSomes (blue) (*n* = 3, technical replicates).

The PSomes in this study were synthesized via photoinitiated RAFT mediated PISA in the absence of conventional catalyst or initiator.^[^ ^25^ ^]^ Building on our previous work,^[^ ^12^ ^]^ we targeted a microliter volume synthetic approach to enable a cost‐effective synthesis of protein‐loaded PSomes. This differs from conventional PISA methods as well as other PSome forming techniques, which require relatively high amounts of proteins due to their milliliter‐scale fabrication process. Key to this reaction miniaturization is endowing visible light initiated (*λ_max_* = 405 nm) RAFT polymerization with oxygen tolerance^[^ ^13^, ^26^ ^]^ and thereby allow polymerization to occur inside unsealed 1546‐well plates. This is achieved by minimizing oxygen diffusion into the polymerization solution with a passivating layer of mineral oil and thereby reducing the inhibition effects typically seen in radical polymerization. To synthesize pH‐responsive PSomes, we chain extended 4‐cyano‐4‐[(dodecylsulfanylthiocarbonyl)sulfanyl]pentanoic acid modified poly(ethylene glycol) monomethyl ether (PEG‐CDTPA) with 2‐hydroxypropyl methacrylate (HPMA) and minor amounts (10 mol% relative to HPMA) of 2‐(dimethylamino)ethyl methacrylate (DMAEMA) as a pH‐responsive co‐monomer yielding PEG‐*b*‐P(HPMA‐*co*‐DMAEMA) (**Figure** **2**A). To form structures that are stable in the extracellular physiological environment, these 10 µL syntheses were all performed in HEPES buffer at pH 7.3. We also synthesized PEG‐*b*‐PHPMA‐based PISA assemblies to serve as a control which would not be expected to display pH responsive behavior (Figure S1, Supporting Information). In both cases, shifts in the molecular weight distributions obtained by gel permeation chromatography (GPC) when compared to the parent PEG‐CDTPA, suggested successful synthesis of diblock copolymers (PEG‐*b*‐PHPMA: *M_n_* = 98 200 g mol^−1^, *Đ* = 1.4; PEG‐*b*‐P(HPMA‐*co*‐DMAEMA): *M_n_* = 120 200 g mol^−1^, *Đ* = 1.6) (Figure S2, Supporting Information). A small low molecular weight peak similar to the elution time of PEG‐CDTPA is seen in the molecular weight distributions of both crude PEG‐*b*‐PHPMA and PEG‐*b*‐P(HPMA‐*co*‐DMAEMA) copolymers which is in line with our previous observation of some active chain loss to oxygen termination events in a non‐deoxygenated setup.^[^ ^12^ ^]^ Regardless, these PEG‐like polymer chains as well as residual monomer could be readily removed by purification through three repeated cycles of centrifugation as confirmed by GPC. (Figure S2, Supporting Information). Unless otherwise stated, all subsequent nanoparticle characterizations were performed after purification by centrifugation.

Having confirmed the successful formation of diblock copolymers, the purified assemblies were subsequently visualized under cryogenic transmission electron microscopy (cryo‐TEM) which in both cases confirmed the formation of unilamellar vesicular structures (PSomes) with membrane thicknesses of 27 ± 6 nm for PEG‐*b*‐PHPMA and 26 ± 6 nm for PEG‐*b*‐P(HPMA‐*co*‐DMAEMA), respectively (Figure 2B,C; Figure S3, Supporting Information) consistent with previous reports of conventionally synthesized HPMA‐based polymersomes.^[^ ^27^ ^]^ Dynamic light scattering (DLS) measurements of the dispersions indicated similar hydrodynamic diameters (D_H_) between the two types of purified PSomes in PBS, with an intensity‐based average size of 417 ± 86 nm for PEG‐*b*‐PHPMA PSomes and 412 ± 79 nm for PEG‐*b*‐P(HPMA‐*co*‐DMAEMA) PSomes as well as narrow polydispersity indexes (PDIs) of 0.05 and 0.04, respectively (Figure 2D,E). This average was found to closely align with the particle sizes obtained via measurement from cryo‐TEM images with diameters of 412 ± 95 nm for PEG‐*b*‐PHPMA and 409 ± 82 nm for PEG‐*b*‐P(HPMA‐*co*‐DMAEMA), respectively.

To investigate the pH‐responsive behavior of the PSomes, we employed DLS to study changes in the PSome population as they were subjected to mildly acidic conditions mimicking the endosomal microenvironment (Figure 2F,G; Section S1 and Figure S4, Supporting Information). As expected, the hydrodynamic diameters for PEG‐*b*‐PHPMA PSomes, which lack readily protonatable groups, remained constant across the pH range of 7.4 to 5.5 (Figure 2F). This was also reflected in the derived count rates (scattering intensity indicative of particle concentration at a constant size) which were also unaffected by pH changes, confirming stability of the system. In contrast, PEG‐*b*‐P(HPMA‐*co*‐DMAEMA) PSomes exhibited a sudden decrease in their hydrodynamic diameter as the pH was lowered from 7.4 to below 6.5 (Figure 2G). In addition, the derived count rate of the PEG‐*b*‐P(HPMA‐*co*‐DMAEMA) PSomes was reduced by approximately two orders of magnitude (from 10^5^ kcps in PBS to 10^3^ kcps at pH 5.5). This indicates that under acidic conditions, the tertiary amines in PEG‐*b*‐P(HPMA‐*co*‐DMAEMA) become protonated as the pH approaches 5.5. Thus, protonation within the hydrophobic core‐forming block of the PSome results in a disruption to the hydrophobic‐hydrophilic balance, which induces PSome disassembly. To test this hypothesis, the surface charge of both types of polymer nanoparticles was probed at different pH values. As expected, owing to the absence of pH‐sensitive motifs in PEG‐*b*‐PHPMA, these PSomes remained neutral when transitioning from pH 7.4 (PBS) to pH 5.5 due to the presence of stabilizing PEG chains. In contrast, while PEG‐*b*‐P(HPMA‐*co*‐DMAEMA) PSomes were neutral in PBS, as the pH was lowered, they acquired an increasing positive surface charge (Figure 2H), consistent with protonation of tertiary amine moieties under acidic conditions. This is in agreement with literature discussing the transition of surface charge toward a positive state in tertiary amine‐functionalized PSomes upon exposure to an acidified environment.^[^ ^28^ ^]^ Taken together, the results from DLS and zeta potential measurements allow classification of the PEG‐*b*‐P(HPMA‐*co*‐DMAEMA) PSomes as pH‐responsive PSomes with PEG‐*b*‐PHPMA PSomes being referred to as inert PSomes.

### 2.2 Loading and Release of Proteins from pH‐Responsive PSomes

As the PSomes synthesized here are fabricated using a PISA approach, macromolecules such as proteins can be readily loaded by addition to the initial reaction mixture and then allowing the PSome membrane to form around them analogous to thin‐film rehydration and other approaches.^[^ ^29^ ^]^ However, when this approach is combined with a low volume, chemical self‐assembly approach as developed here, higher throughput formulation of protein‐loaded nanoparticles can be achieved as reaction parallelization is greatly facilitated at microliter volume scales. To demonstrate this, we studied the loading of a variety of model proteins (with a range of molecular weights from 28 to 160 kDa and isoelectric points from 4.2 to 9.5) by synthesizing multiple wells of protein‐loaded PSomes directly in a 1536‐well plate and at a reaction volume of 10 µL. After purification by centrifugation, bulk measurements of their respective protein encapsulations were then quantified by a modified MicroBCA assay, revealing encapsulation efficiencies ranging from 5 to 30% which appears dependent on both the nature of the protein itself as well as the initial protein concentration (Figure S5, Supporting Information). For example, BSA loaded in this fashion achieved encapsulation efficiencies of 9.9 ± 4.1% for inert PSomes and 10.2 ± 1.5% for pH‐responsive PSomes when added to the pre‐polymerization mixture at a concentration of 1 mg mL^−1^. These values are in broad agreement with our previous study of PEG‐*b*‐PHPMA polymersomes with loaded *Gaussia* luciferase (GLuc).^[^ ^12^ ^]^ Similar to previous literature,^[^ ^30^ ^]^ polymersomes loaded with different proteins presented relatively similar sizes to unloaded polymersomes according to DLS and importantly, retained their pH responsive behavior (Figure S6, Supporting Information). Considering its well‐characterized properties, BSA was chosen as a model protein for further polymersome characterization and intracellular delivery experiments.

**Figure 3:**
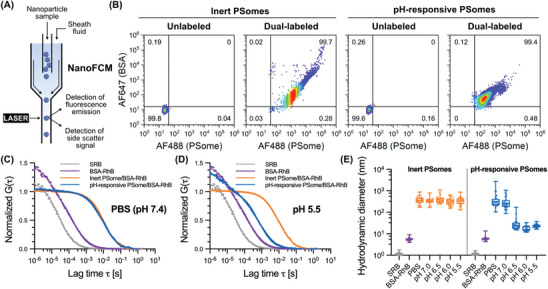
Single particle characterization of protein loading and release from PSomes. A) Schematic representation of a NanoFCM experiment. B) NanoFCM‐derived bivariate dot‐plots of AF647 (BSA) versus AF488 (PSome) for unlabeled and dual‐labeled PSome‐AF488/BSA‐AF647 using inert or pH‐responsive PSomes (*n* = 4000‐10000 particles). Normalized FCS autocorrelation curves of sulforhodamine B (SRB), BSA‐RhB, inert PSome/BSA‐RhB, and pH‐responsive PSome/BSA‐RhB in C) PBS and D) pH 5.5 buffer (average curves of *n* = 25 individual measurements, dots represent raw data, lines are fitted curves). E) Hydrodynamic diameters calculated from FCS autocorrelation analysis for inert PSome/BSA‐RhB (orange) and pH‐responsive PSome/BSA‐RhB (blue) at various pH values (*n* = 25 individual measurements). Box plots: center line, median; box limits, upper and lower quartiles; whiskers, minimum and maximum values.

Important to note is that conventional methods for characterizing cargo loading within nanoparticles, such as the MicroBCA assay, high‐performance liquid chromatography (HPLC), or absorption/fluorescence spectroscopy of the unloaded cargo, are limited to yielding bulk (and sometimes indirect) information of the particle population, providing limited insights into cargo loading. A useful tool in this regard to study the loading of macromolecules on a single‐particle basis is nanoparticle flow cytometry (NanoFCM), which is a highly sensitive form of conventional flow cytometry but for nanoparticles. This single‐particle analytical technique measures the side scattering and fluorescence emission of particles as they flow through a laser source within a directed fluid stream allowing measurements of individual particles within nanoparticle populations (**Figure** **3**A).^[^ ^31^ ^]^ As such, NanoFCM can be a powerful technique for studying the loading of macromolecules into PSomes and can even enable examination of the heterogeneity of loading. We therefore loaded BSA as a model protein into dual‐labeled polymersomes, with separate fluorescent labels for the polymer and protein (PSomes‐AF488/BSA‐AF647). This was achieved by the addition of a small amount of an azide‐functionalized monomer (AzHPMA) to the synthesis mixture also containing Alexa Fluor 647‐labeled bovine serum albumin (BSA‐AF647), followed by post‐polymerization conjugation of the polymer chains with AF488‐DBCO using strain promoted azide‐alkyne cycloaddition (SPAAC). After removal of free dyes and proteins by centrifugation, NanoFCM revealed that for both inert and pH responsive PSomes, over 95% of the particle population exhibited signals for both AF488 and AF647 (Figure 3B; Figure S7, Supporting Information). The association of BSA‐AF647 with the AF488‐labeled PSomes points strongly toward the encapsulation of BSA‐AF647 within the PSomes, as no particles with AF647 fluorescence were detected in physical mixtures of BSA‐AF647 and pre‐formed empty PSomes (Figure S8, Supporting Information) suggesting the importance of protein loading during the PISA process. Additionally, the PSomes were stable and protected their encapsulated cargo (glucose oxidase as a model enzyme) against external degrading agents, such as proteases, even after 7 days of incubation at 37 °C and physiological pH (Section S2 and Figure S9, Supporting Information). This protective effect against proteases of PISA derived ensembles encapsulating enzymes/proteins is in agreement with previous literature.^[^ ^11^, ^32^ ^]^ These results confirm an encapsulation mechanism for protein cargo within the aqueous lumen of PISA‐derived PSomes, and that the pH‐responsive PSome membrane maintains its protective barrier against proteolytic degradation when kept under physiological pH and temperature.

Having demonstrated the successful encapsulation of proteins within DMAEMA‐functionalized PSomes that disassemble under acidic conditions, we sought to investigate whether the encapsulated contents could be released under these varying pH conditions. Therefore, FCS was employed to analyze the diffusion of fluorescently labeled species in solution, enabling the discrimination of smaller particles with higher diffusivity from larger particles with lower diffusivity.^[^ ^33^ ^]^ To study protein release triggered by variations in pH, the diffusion profile of rhodamine B‐labeled BSA (BSA‐RhB) loaded within PSomes (PSome/BSA‐RhB) was studied across pH values from 7.4 to 5.5. FCS autocorrelation curves (Figure 3C,D; Figure S10A‐C, Supporting Information) revealed that inert PSome/BSA‐RhB maintained a constant diffusion time distinctively longer than that of free BSA‐RhB across the pH range tested, indicating retention of the protein within the PSomes. In contrast, pH‐responsive PSome/BSA‐RhB showed a clear shift toward faster diffusion times upon acidification, indicative of release of BSA‐RhB from the larger, slower diffusing polymersome. This is reflected when calculating effective hydrodynamic diameters, whereby pH‐responsive PSome/BSA‐RhB presented a similar diameter to inert PSomes when under physiological conditions; however, only pH‐responsive PSome/BSA‐RhB showed a drastic decrease in effective diameter upon decreasing the pH toward 5.5 (Figure 3E). This result was strongly mirrored in the DLS measurements taken of the two types of PSomes (Figure 2F,G). Importantly, FCS also enables measurements of the number of BSA‐RhB per PSome (calculated via molecular brightness values, cpp) which supports the finding that the pH‐responsive system disassembles upon acidification, while the inert PSomes remain stable and keep the protein encapsulated (Figure S10D, Supporting Information). The slightly higher hydrodynamic diameter and cpp values of released BSA‐RhB compared to free BSA‐RhB indicates an electrostatic and/or hydrophobic association of the protein with some polymer chains upon disassembly, as might be expected in a buffer‐only environment that lacks other competing components that could disturb this interaction further.^[^ ^34^ ^]^ Overall, these findings suggest that the protein loaded within pH‐responsive PSomes can be controllably released at pH values lower than 6.5 and indicates the potential for delivery of macromolecular cargo via the acidified endolysosomal pathway.

### 2.3 pH‐Responsive PSomes for Intracellular Protein Delivery

Having demonstrated suitable pH‐responsive release of encapsulated proteins, we sought to apply this ultralow volume platform for studying intracellular delivery. To elucidate this, the cytocompatibility of the PSomes was assessed across a range of polymer concentrations against MCF‐7 cells as a model cell line. Using an MTS assay, both inert and pH‐responsive PSomes showed no loss of cell viability after 24 h of incubation with up to 2 mg mL^−1^ of polymer, indicating that the PSome carrier alone does not induce toxicity (Figure S11, Supporting Information). We then evaluated the cell uptake of the protein‐loaded PSomes by conducting flow cytometry (FCM) analysis. After incubation with dual‐labeled PSome‐AF488/BSA‐AF647 for 24 h, over 85% of MCF‐7 cells were doubly positive for both AF488 (PSome) and AF647 (BSA) fluorophores compared to untreated cells (Figure S12, Supporting Information). The mechanism of cellular internalization was investigated further (Section S3 and Figure S13, Supporting Information), with uptake inhibitors revealing that the PSomes were uptaken primarily via energy‐dependent endocytosis with macropinocytosis and clathrin‐mediated endocytosis as dominant uptake pathways.

**Figure 4:**
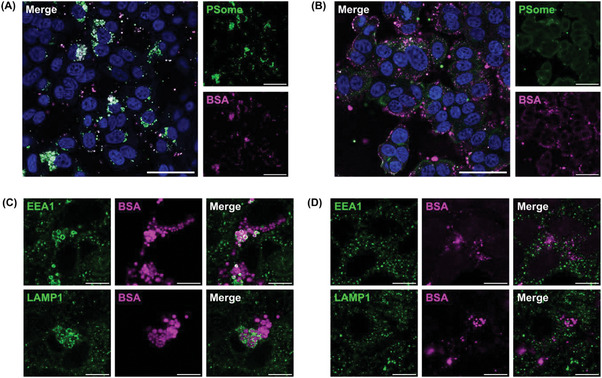
Cellular uptake of BSA loaded PSomes in MCF‐7 cells. Representative confocal microscopy images of MCF‐7 cells treated with A) dual‐labeled inert PSome‐AF488/BSA‐AF647 and B) dual‐labeled pH‐responsive PSome‐AF488/BSA‐AF647 for 24 h. For confocal microscopy, nuclei were stained with Hoechst 33342 (blue), PSomes with AF488 (green), and encapsulated BSA with AF647 (magenta). Merged images represent the overlay of the three separate channels. Scale bars: 50 µm. Immunofluorescent staining of endolysosomal compartments within MCF‐7 cells after incubation with either C) inert PSomes with loaded BSA‐AF647 or D) pH‐responsive PSomes with loaded BSA‐AF647 for 4 h. Endosomes or lysosomes (green) were stained using mouse anti‐EEA1 monoclonal antibody for early endosomes or rabbit anti‐LAMP1 monoclonal antibody for lysosomes, followed by labeling with a secondary AF555 conjugated antibody. Merged images represent the overlay of the two separate channels. Scale bars: 10 µm.

Having demonstrated successful internalization of protein‐loaded PSomes by MCF‐7 cells, we aimed to assess their ability to perform intracellular protein delivery by exploiting the endolysosomal microenvironment and the pH‐mediated release mechanism of the DMAEMA functional PSomes. We initially validated the intracellular colocalization of PSome‐AF488 and BSA‐AF647 signals within MCF‐7 cells using confocal laser scanning microscopy (CLSM). After 24 h of incubation, distinct intracellular colocalization of both fluorophores was evident in MCF‐7 cells treated with dual‐labeled inert PSome‐AF488/BSA‐AF647 (**Figure** **4**A). This is consistent with our previous report on the stability of macromolecules loaded within PEG‐*b*‐PHPMA PSomes which displayed strongly colocalized signals for at least 7 days after initial uptake in another cell type.^[^ ^12^ ^]^ When cells were instead treated with dual‐labeled pH‐responsive PSomes, generally more diffuse BSA signals were observed along with poor fluorophore colocalization, suggesting displacement of the encapsulated protein from the PSome carrier **(Figure** 4B). Such observations mirror the behaviors of the well‐known calcein leakage assay whereby punctuate fluorescent signals from endosomally trapped calcein become more diffuse if calcein can escape the endosomal compartment and distribute into the intracellular space.^[^ ^35^ ^]^ The lack of colocalization between BSA‐AF647 and pH responsive PSome‐AF488 was also confirmed by determination of the Mander's overlap coefficient^[^ ^36^ ^]^ which indicated significantly lower colocalization of labeled protein and pH‐responsive PSomes (R = 0.14 ± 0.06) when compared to proteins loaded within inert PSomes (R = 0.89 ± 0.03) (Figure S14, Supporting Information). Overall, these results suggest the potential for pH‐responsive PSomes to release proteins intracellularly, whereas proteins encapsulated within inert PSomes remain tightly associated with the PSome carrier after uptake.

### 2.4 Endosomal Escape of Protein‐Loaded pH‐Responsive PSomes

To gain insight into the mechanism underlying intracellular protein delivery facilitated by the pH‐responsive PSomes, an investigation into the intracellular trafficking of protein‐loaded PSomes within MCF‐7 cells was conducted using CLSM. Endosomal marker labeling was performed by immunofluorescent staining with antibodies against early endosome antigen 1 (EEA1) and lysosomal‐associated membrane protein 1 (LAMP1). EEA1 identifies early endosomal compartments, while LAMP1 is a marker of late endosomes and lysosomes. Confocal images of cells incubated with dual‐labeled inert PSome‐AF488/BSA‐AF647 for 4 h showed colocalization of endosomal markers (green) and BSA‐AF647 (magenta) fluorescence (Figure 4C). In contrast, when the pH‐responsive PSomes were employed as a carrier, there was negligible colocalization of BSA‐AF647 (magenta) with EEA1 and LAMP1‐positive vesicles (green) (Figure 4D), suggesting endosomal release of the protein. We hypothesize that this release occurs in response to the decrease in pH as the endosomes progress through different stages of maturation (pH 6.0 – 4.9).^[^ ^19^ ^]^ Importantly, these results provide evidence for endolysosomal escape and cytosolic localization of protein cargo occurring only when pH‐responsive PSomes are employed as a carrier.

Since the direct visualization of intracellular compartments can be restricted by the resolution limit of confocal microscopy (≈250 nm),^[^ ^37^ ^]^ we conducted further mechanistic investigations of endosomal escape using STORM.^[^ ^38^ ^]^ This is a super‐resolution fluorescence imaging technique with a lateral resolution of ≈20 nm that determines the precise localization of individual stochastically blinking fluorescent molecules. By allowing for sub‐diffraction limit imaging, STORM is particularly powerful for the study of intracellular trafficking and has recently been applied to visualize the leakage of cargo from swollen or ruptured lysosomes.^[^ ^24^, ^39^ ^]^

**Figure 5:**
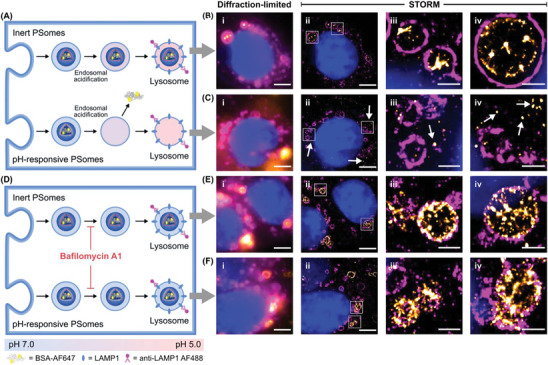
Two‐color super resolution STORM images of BSA‐loaded PSomes and endolysosomal compartments with and without treatment with BafA1. Schematic illustration of STORM analysis for MCF‐7 cells incubated with BSA‐AF647‐loaded inert and pH‐responsive PSomes A) without and D) with BafA1 pre‐treatment. Representative images of MCF‐7 cells treated with B, E) BSA‐loaded inert PSomes or C,F) BSA‐loaded pH‐responsive PSomes for 4 h. Cells were pre‐treated with (E,F) or without (B,C) BafA1 for 1 h prior to incubation with PSomes. Nuclei were stained with DAPI (blue), the late endosomes/lysosomes were stained with AF488‐labeled anti‐LAMP1 (magenta), and the BSA was labeled with AF647 (yellow) as a model protein. For clarity, white arrows have been added to indicate areas whereby AF647‐BSA is identified outside the late endosomes/lysosomes. For images in B, C, E, and F: (i) diffraction‐limited images; (ii) STORM reconstructed images; (iii – iv) zoom‐in of STORM reconstructed images. Scale bars (i – ii): 5 µm. Scale bars (iii – iv): 1 µm.

To this end, MCF‐7 cells were treated with BSA‐AF647‐loaded inert PSomes or BSA‐AF647‐loaded pH‐responsive PSomes for 4 h. To visualize the lysosomes, the cells were then fixed and immunostained for LAMP1 with AF488 (**Figure** **5**A). The association between LAMP1‐positive vesicles and the loaded proteins was then visualized by STORM. Representative STORM images of cells treated with BSA‐AF647‐loaded inert PSomes showed BSA‐AF647 largely entrapped within LAMP1‐positive vesicles (Figure 5B), whereas cells treated with BSA‐AF647‐loaded pH‐responsive PSomes exhibited much poorer overlap between lysosomal vesicles and BSA‐AF647 (Figure 5C). This was accompanied by diffuse BSA‐AF647 signals outside the late endosomal/lysosomal vesicles (Figure 5Ciii‐iv, white arrows). Together, these results are consistent with the observation of intracellular localization of protein cargo by confocal microscopy (Figure 4C,D) and suggest that only protein cargo delivered within pH‐responsive PSomes (not inert PSomes) can be successfully released from late endosomes and lysosomes. Similarly, when treated with PSomes for a longer incubation time of 24 h, we also observed the entrapment of BSA‐AF647 within LAMP1‐positive lysosomes in cells treated with BSA‐647 loaded inert PSomes but not pH‐responsive PSomes (Figure S15, Supporting Information).

To further probe this behavior, STORM was also conducted after labeling the polymer chains with AF488 to visualize their interaction with the lysosomal marker (Figure S16, Supporting Information). Consistent with protein‐labeled experiments (Figure 5B; Figure S15, Supporting Information), STORM images of AF488‐labeled inert PSomes revealed the majority of PSome‐AF488 signals to be entrapped within LAMP1‐positive vesicles (Figure S16B, Supporting Information). Intriguingly, AF488‐labeled pH‐responsive PSomes showed a distinct pattern whereby AF488 signals were closely associated with the lysosomal membrane (Figure S16C, Supporting Information), suggesting an interaction of the protonated polymer with the negatively charged lysosomal membrane as observed by others.^[^ ^40^ ^]^

As we hypothesized that endosomal escape was linked to the pH‐dependent release mechanism of pH‐responsive PSomes, we employed bafilomycin A1 (BafA1) as a V‐ATPase inhibitor to inhibit acidification of endosomal vesicles (Figure 5D).^[^ ^41^ ^]^ To this end, cells were pre‐treated with and without BafA1 for 1 h prior to incubation with BSA‐AF647‐loaded inert and pH‐responsive PSomes for 4 h. In contrast to the previous uptake and trafficking observations, pre‐treatment with BafA1 resulted in distinct colocalization of BSA‐AF647 with lysosomal vesicles regardless of whether BSA‐AF647 was loaded within pH‐responsive or inert PSomes (Figure 5E,F). The retention of BSA‐AF647 within non‐acidified LAMP1‐positive vesicles, particularly in the context of pH‐responsive PSomes, suggests that the earlier observations of endosomal escape are related to a proton sponge‐like mechanism however further investigations may be warranted.^[^ ^40^, ^42^ ^]^

Finally, to demonstrate that the delivered protein retains its activity in the intracellular space, we also fabricated PSomes loaded with horseradish peroxidase (HRP) as a model enzyme capable of producing a colorimetric signal after cellular uptake. Interestingly, despite the presence of the heme moiety within HRP, the enzyme retained essentially all of its initial activity under the photopolymerization conditions (Figure S17, Supporting Information). Following 24 h incubation of MCF‐7 cells with HRP‐loaded polymersomes and subsequent removal of non‐uptaken PSomes, an in vitro TMB assay was conducted to assess peroxidase activity within the MCF‐7 cells (Figure S18, Supporting Information). Untreated cells or cells treated with free HRP or empty PSomes exhibited little TMB activity indicating a lack of free HRP uptake when unformulated as well as negligible background peroxidase activity by the cells or empty PSomes. In contrast, significant peroxidase activity was observed after treatment of cells with HRP‐loaded PSomes indicating that protein functionality can be retained after cellular internalization, regardless of endosomal entrapment or intracellular release (Figure S18, Supporting Information).

### 2.5 Delivery of Functional Proteins for the Activation of Dendritic Cells

Having probed the mechanism behind the efficient intracellular delivery of proteins, we sought to exploit this platform as a proof of concept to elicit a functional response in a model system. We hypothesized that the developed pH‐responsive PSome platform could be particularly potent for enhancing the intracellular delivery of macromolecular antigens to antigen‐presenting cells (APCs) such as dendritic cells (DCs) for applications in protein subunit vaccines and immunotherapy. Such applications benefit greatly from the ultralow volume PSome encapsulation approach developed here, which could allow for the high throughput in vitro screening of antigens without laborious scaled‐up protein production.

**Figure 6:**
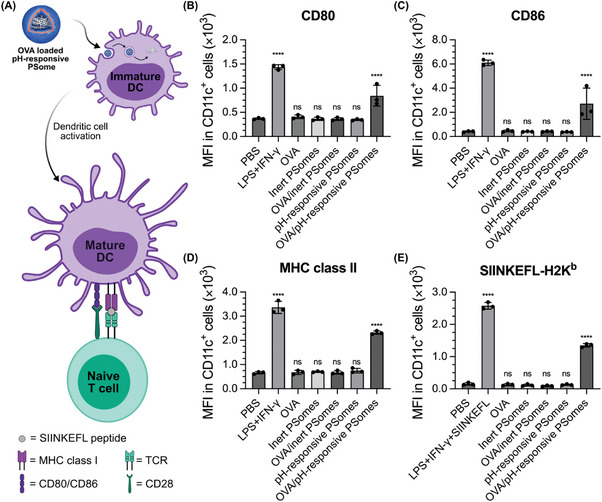
In vitro activation of BMDCs and antigen cross‐presentation mediated by OVA‐loaded pH‐responsive PSomes. A) Schematic illustration of OVA‐loaded pH‐responsive PSomes enhancing maturation and antigen cross‐presentation in dendritic cells. Characterization of surface markers B) CD80, C) CD86, D) MHC class II, and E) SIINKEFL (OVA (257‐264)) bound to MHC class I by immunoflow cytometry analysis under various treatments in CD11c^+^ cells. Cells were stained with antibodies against CD11c as the DC marker, CD80, CD86, and MHC class II as DC maturation markers as well as SIINKEFL‐H2K^b^ to measure OVA antigen cross‐presentation. Data presented as mean ± SD (*n* = 3, technical replicates). Statistical significance was determined using one‐way ANOVA with Dunnett's multiple comparisons test. ****p < 0.0001; *ns* not significant.

Protein‐based vaccines function by facilitating uptake of foreign antigens while stimulating innate danger sensors to initiate APC maturation, resulting in the upregulation of antigen presentation machinery (e.g., MHC class I and II) and co‐stimulatory molecules (e.g., CD80 and CD86) to activate antigen‐specific adaptive immunity (**Figure** **6**A). While nanoparticle formulations are known to enhance APC uptake of protein antigens compared to soluble protein, it has been suggested that pH‐sensitive delivery systems may further enhance vaccine function by promoting antigen entry into the cytosolic processing pathway and by releasing danger‐associated molecular patterns (DAMPs) or activating the NLRP3 inflammasome during endosomal/lysosomal disruption.^[^ ^43^ ^]^ Thus, we hypothesized that pH‐responsive PSomes can simultaneously promote efficient antigen uptake, enhance antigen cross‐presentation on MHC class I, and stimulate innate activation of APCs, functions that are essential for priming potent cytotoxic T‐cell responses in therapeutic cancer vaccines.^[^ ^44^ ^]^

To evaluate the impact of pH‐responsiveness on PSome‐mediated DC maturation and antigen cross‐presentation, we loaded pH‐responsive and inert PSomes with OVA, a widely studied model antigen with low soluble uptake that is cross‐presented efficiently if delivered to the cytoplasm.^[^ ^43^, ^45^ ^]^ OVA‐loaded PSomes were then exposed to BMDCs and their ability to induce cellular activation was investigated for surface expression of CD80, CD86, MHC class I, and MHC class II via flow cytometry. Immature BMDCs, when incubated with lipopolysaccharide (LPS) and interferon‐gamma (IFN‐γ), exhibited significant 5‐, 14‐, and 6‐fold increases in CD80, CD86, and MHC class II expression, respectively, compared to BMDCs treated with PBS, aligning with the expected maturation of BMDCs upon potent innate stimulation (Figure 6B–D). In contrast, immature BMDCs treated with free OVA antigen revealed an inability to initiate DC maturation presumably due to poor cellular uptake of the antigen alone.^[^ ^46^ ^]^ OVA‐loaded, but not empty, pH‐responsive PSomes promoted BMDC maturation (2‐, 7‐, and 4‐fold increase in CD80, CD86, and MHC class II expression), indicating that PSomes provide little intrinsic adjuvant activity but facilitate cellular uptake and intracellular antigen release. As expected, both empty and OVA‐loaded inert PSomes induced negligible expression of CD80, CD86 and MHC class II, consistent with stable entrapment of OVA within the PSome.

Encouraged by the successful activation of BMDCs by OVA‐loaded pH‐responsive PSomes, we next assessed cross‐presentation efficiency, which was quantified by flow cytometry measurements of SIINFEKL bound to surface H‐2K^b^ (MHC class I). Activating BMDCs with LPS and IFN‐γ and pulsing with the OVA(257‐264) fragment (SIINFEKL) resulted in a 17‐fold increase in SIINKEFL bound to MHC class I when compared to BMDCs treated only with PBS, consistent with known TLR‐enhancement of cross presentation.^[^ ^47^ ^]^ In contrast, BMDCs treated with free OVA, empty PSomes, or OVA‐loaded inert PSomes showed negligible presentation of SIINFEKL on the MHC class I molecules (Figure 6E). Importantly, treating BMDCs with OVA‐loaded pH‐responsive PSomes resulted in 8‐fold increases in SIINFEKL cross‐presentation, indicating the key role of the pH‐responsive PSome in facilitating intracellular OVA delivery for efficient antigen processing within BMDCs. This marked enhancement in SIINFEKL cross‐presentation demonstrates the utility of pH‐responsive PSomes in facilitating antigen presentation and APC maturation and is highly promising as an in vitro screening platform for immune modulation and vaccine applications. Future work will focus on investigating the feasibility of these polymersomes for delivery of clinically‐relevant antigens and adjuvants.

## 3 Conclusion

In this work, we have presented a microliter volume scale synthetic platform for producing protein‐loaded pH‐responsive PSomes via an oxygen tolerant Photo‐PISA process. These PSomes are highly stable, can load a variety of proteins and can protect encapsulated proteins for at least 7 days under proteolytic conditions in vitro. The PSomes were extensively characterized on a single particle basis allowing for the study of the mechanism of protein encapsulation and loading heterogeneity by NanoFCM as well as triggerable protein release under acidic conditions using FCS. We demonstrated that this acidic release mechanism can be directly exploited to trigger intracellular protein delivery via escape from the late endosomal and lysosomal compartment, as confirmed by CLSM and super‐resolution STORM imaging in MCF‐7 cells. Antigen‐loaded pH‐responsive PSomes further demonstrated effective intracellular delivery and antigen‐dependent stimulation of primary BMDCs without material‐mediated adjuvant effects. This delivery system thus has excellent potential as a general biomacromolecule delivery system and should be further evaluated with additional cargoes for applications in vaccines and cancer immunotherapy. By producing this delivery system with a microliter scale fabrication process, we unlock the potential to screen otherwise cost‐prohibitive or early‐stage therapeutics which are difficult to source or manufacture in large quantities for preclinical drug discovery research. Considering the remarkable versatility and effectiveness of this platform for intracellular macromolecular delivery, we envision it will have profound implications for a range of therapeutic and biotechnological applications.

## 4 Experimental Section

### Materials

Poly(ethylene glycol) monomethyl ether (mPEG_113_, *M_n_* = 5 000 g mol^−1^, Sigma‐Aldrich), 4‐cyano‐4‐[(dodecylsulfanylthiocarbonyl)sulfanyl] pentanoic acid (CDTPA, Boron Molecular), *N*,*N*′‐dicyclohexylcarbodiimide (DCC, Sigma‐Aldrich), 4‐(dimethylamino)pyridine (DMAP, Sigma‐Aldrich), mineral oil (BioReagent, Sigma‐Aldrich), and all other reagents were used as received unless otherwise specified. mPEG_113_ was terminally modified with CDTPA, yielding PEG_113_‐CDTPA, according to a published protocol.^[^ ^48^ ^]^ HPMA was purified to remove impurities by silica column chromatography using ethyl acetate:hexane (1:9) as eluent. DMAEMA was purified by passing the monomer through a column filled with basic aluminium oxide to remove the inhibitor. 3‐azido‐2‐hydroxypropyl methacrylate (AzHPMA) was synthesized as previously reported.^[^ ^49^ ^]^ All vinyl monomers were stored at −20 °C. Dyes for labeling: Alexa Fluor 488‐DBCO (AF488‐DBCO, Jena Bioscience), AZDye 647 NHS ester (AF647‐NHS, Fluoroprobes) and rhodamine B isothiocyanate (RhB‐ITC, Merck) were prepared as 10 mM stock solutions in DMSO and stored at −20 °C prior to use. Bovine serum albumin (BSA), glucose oxidase from *Aspergillus niger* (GOx), ovalbumin (OVA), peroxidase from horseradish (HRP), catalase from bovine liver, Saporin from *Saponaria officinalis* seeds (SAP), 3,3′,5,5′‐tetramethylbenzidine (TMB), Triton X‐100, chlorpromazine hydrochloride (CPZ), 5‐(N‐ethyl‐N‐isopropyl)amiloride (EIPA), and Filipin III from *Streptomyces filipinensis* were purchased from Sigma‐Aldrich. HEPES buffer, Tris‐HCl buffer, Halt Protease Inhibitor Cocktail (100X), DMEM medium with 4.5 g L^−1^ glucose and GlutaMAX, RPMI 1640 medium, heat‐inactivated fetal bovine serum (FBS), Penicillin‐Streptomycin (10 000 U mL^−1^), 2‐mercaptoethanol (β‐ME), ACK lysis buffer, 0.05% (w/v) Trypsin‐EDTA with phenol red, TrypLE Express Enzyme, 4% (w/v) paraformaldehyde (PFA) in PBS, goat anti‐mouse IgG1 Cross‐Adsorbed Secondary Antibody conjugated with Alexa Fluor 555, goat anti‐rabbit IgG (H+L) Cross‐Adsorbed Secondary Antibody conjugated with Alexa Fluor 555, Hoechst 33342 Solution (20 mM), and SlowFade Diamond Antifade Mountant were obtained from Thermo Fisher Scientific. Recombinant murine Granulocyte‐Macrophage Colony‐Stimulating Factor (GM‐CSF) was purchased from PeproTech.

### Instrumentation

All photopolymerizations were conducted by using a Teleopto LAD‐1 LED array driver powering a LEDA‐V (*λ_max_* = 405 nm) LED array with a light intensity of ≈10 mW cm^−2^. The reaction was performed in a clear, flat bottom, HiBase, 1536‐well polystyrene plate (Greiner Bio‐One, Austria) with the LED array on the top of the microtiter plate.

For PSome characterization, molecular weight distributions and dispersity (*Ð*) of the block copolymers were obtained from GPC using a 1260 Infinity II GPC MDS (refractive index detection only) equipped with a PSS GRAM guard column (8 × 50 mm, 10 µm) and two PSS GRAM linear columns (8 × 300 mm, 10 µm, 500–1 000 000 Da). The eluent was HPLC grade DMF containing 0.075% (w/w) LiBr and was run at a flow rate of 1 mL min^−1^ at 40 °C. Molecular weight calibration was performed using near‐monodisperse poly(methyl methacrylate) standards (EasiVial, Agilent).

DLS measurements were performed in technical triplicates using a Malvern ZetaSizer Nano ZS. Size measurements of the PSomes were performed at a polymer concentration of 1 mg mL^−1^ in different pH buffers: PBS (pH 7.4), 10 mM phosphate buffer (pH 7.0, pH 6.5, pH 6.0), and 10 mM acetic acid/sodium acetate (pH 5.5). Zeta potential measurements were conducted with PSomes (0.02 mg mL^−1^) in 50x diluted PBS (pH 7.4), 2 mM phosphate buffer (pH 7.0, pH 6.5, pH 6.0), and 2 mM acetic acid/sodium acetate (pH 5.5).

For cryogenic transmission electron microscopy (cryo‐TEM), 3.0 µl of PSome dispersions (1 mg mL^−1^) in DPBS were applied to freshly glow‐discharged Lacey carbon grids (EM Resolutions, UK), blotted for 5 sec, and plunged into liquid ethane, using a Leica GP2 (Leica, Germany) automated plunge‐freezer operated at 4 °C and 95% relative humidity. The cryo‐specimens were transferred to a JEOL 2100Plus microscope operated at 200 kV, using a Gatan 626 cryo‐holder. Images were recorded on a Gatan OneView camera. Membrane thicknesses and diameters were calculated as mean ± SD using the line drawing tool within the ImageJ software (NIH, USA). The measurements were obtained by analyzing <150 PSomes.

Nanoparticle flow cytometry was performed using a Flow NanoAnalyzer (NanoFCM Inc, UK) equipped with 488 nm and 638 nm lasers and photodiode detectors on three channels with 488/15, 525/40, and 670/30 bandpass filters. The laser power was set at 10 mW and 20 mW for the 488 nm and 638 nm lasers, respectively, with 0.2% SS decay. The PSomes were diluted 1000 times in PBS prior to the analysis. Single particle signal acquisition was performed for 4 000–10 000 particles with 1 kPa of sampling pressure. PBS was used for background correction of all data presented. The FCS data files were generated through the NanoFCM Professional Suite v1.8 software, and they then were further analyzed in FlowJo software (BD Biosciences).

FCS data acquisition was performed using a commercial LSM 880 (Carl Zeiss, Jena, Germany). A HeNe‐laser (561 nm excitation) and appropriate filter sets were used to obtain the fluorescence intensity fluctuations for single channel configuration. All measurements were carried out at a temperature of 37 °C. The measurements were performed at 200 µm above the glass surface of the glass‐bottom ibidi eight‐well plates using a 40x C‐Apochromat water immersion objective with a numerical aperture (NA) of 1.2. Each sample was measured for 25 × 5 s to obtain the intensity traces. The intensity traces were automatically autocorrelated by ZEN software (Carl Zeiss), and the exported data was fitted using PyCorrFit program 1.1.6,^[^ ^50^ ^]^ employing one component fits (*G*_1*comp*_(τ)).

(1) $$G_{1comp}\left(\tau \right)=\left(1+\frac{T}{1-T}e^{\frac{-\tau }{\tau_{trip}}}\right)*\frac{1}{N*\left(1+\frac{\tau }{\tau_{D}}\right)*\sqrt{1+\frac{\tau }{SP^{2}\tau_{D}}}}$$

Where τ_*D*_ is the diffusion time, τ_*trip*_ is the triplet time (fixed between 1 – 10 µs) of triplet fraction *T*, *N* is the effective average number of diffusing species within the confocal volume, and *SP* is the structural parameter (fixed to 5).

The x‐y dimension of the confocal volume ($\omega_{xy}^{2}$) was calibrated using solutions of sulforhodamine B (SRB) in PBS (D = 5.54 × 10^−6^ cm^2^ s^−1^ at 37 °C, D = 4.14 × 10^−6^ cm^2^ s^−1^ at 25 °C).^[^ ^51^ ^]^ This allowed for the calculation of the diffusion coefficients (*D*) of the experimental samples by using the obtained diffusion times (τ_*D*_) from the autocorrelation fits:

(2) $$D=\frac{\omega_{xy}^{2}}{4\tau_{D}}$$

The hydrodynamic radii (*R_h_*) were then determined using the Einstein‐Stokes equation, using the diffusion coefficients (*D*) obtained earlier.

Flow cytometry measurements were performed using a BD LSRFortessa Cell Analyzer (BD Biosciences). The flow cytometer was equipped with 4 lasers (405 nm, 488 nm, 561 nm, and 640 nm), a fixed optical alignment, and a pressure‐driven fluidics system. All flow cytometry samples were fixed with 4% (w/v) PFA in PBS, and sequentially washed and resuspended in DPBS. Data were analyzed using FlowJo software (BD Biosciences).

Confocal imaging was performed using a Leica SP8 inverted confocal microscope (Leica Microsystems, Germany) with a 63x oil immersion objective lens. Images were processed using Image J software (National Institutes of Health, MD, USA).

STORM was conducted as in Bost et al.^[^ ^24b^ ^]^ In brief, we used a Nikon Ti Eclipse inverted microscope (Nikon, Japan), presenting cube filters (excitation: Chroma ZET405/488/561/640x, emission: Chroma ZET405/488/561/640 m) and TIRF dichroic ZET405/488/561/640bs, equipped with Cairn laser module (Cairn Research, Kent, UK) with 200 mW 488 nm and 140 mW 642 nm lasers used in this study. CFI SR Apo TIRF 100x oil objective (N.A. 1.49) was used, in combination with 1.5x Optovar lens, giving a final magnification of 150x. The camera (Andor iXon Ultra 888 EMCCD, Oxford Instruments, UK) had a pixel size of 13 µm, making the final pixel size 87 nm after the 150x magnification. A 256 × 256 pixels region of interest (ROI) was imaged, and a diffraction‐limited image was acquired for reference of each ROI before starting the STORM acquisition. STORM imaging was conducted with 100% laser power and the acquisition started when steady fluorophore blinking was reached. Each image consisted of 10 000–30 000 frames, with an exposure time of 30 ms per frame. 642 nm and 488 nm channels were imaged sequentially in the STORM mode, whereas the DAPI channel (405 nm) was acquired in diffraction limited mode for each ROI. The final super‐resolved images were reconstructed with the ThunderSTORM plugin in Fiji, followed by drift correction and sigma‐based filtering to remove noise and other low‐quality artefacts.^[^ ^52^ ^]^ A Normalized Gaussian visualization method with magnification of 10x was used for the panel images.

### Synthesis of PEG‐b‐PHPMA

PEG‐*b*‐PHPMA was synthesized using a previously reported protocol.^[^ ^48^ ^]^ PEG_113_‐CDTPA was dissolved in methanol at 10 mg mL^−1^, and 11.38 µL of the stock solution was added to a low protein binding microcentrifuge tube. The solvent was allowed to dry by leaving the tube uncapped at room temperature. HPMA (1.14 µL, 8.43 µmol) was then added to the tube, followed by 8.89 µL of 100 mM HEPES buffer solution at pH 7.3. The pre‐polymerization solution was added to a 1536‐well plate and covered with 2 µL of mineral oil to minimize evaporation during photopolymerization. The plate was irradiated with a 405 nm LED array for 3 h. After photo‐PISA, the crude PSome was collected and diluted with 5X PBS to a final volume of 200 µL. The crude solution was then centrifuged at 14 000 × g for 10 min at room temperature. The supernatant was gently discarded, and the pellet resuspended in 200 µL of fresh 5X PBS. The centrifugation cycle was repeated for two additional cycles. The final pellet from the last centrifugation was then resuspended in 10 µL of DPBS for further analysis.

### Synthesis of PEG‐b‐P(HPMA‐co‐DMAEMA)

A typical synthesis of PEG‐*b*‐P(HPMA‐*co*‐DMAEMA) was as follows. The PEG_113_‐CDTPA aliquots were prepared in an identical protocol as above. HPMA (1.03 µL, 7.59 µmol), DMAEMA (1.42 µL of 93.3 mg mL^−1^ stock solution in 100 mM HEPES buffer, 0.84 µmol) and 7.55 µL of 100 mM HEPES buffer solution at pH 7.3 was added into a tube. The photopolymerization and purification of PSomes was performed as described for the synthesis of PEG‐*b*‐PHPMA.

### Synthesis of Protein‐Loaded PSomes

Protein‐loaded PSomes were fabricated following the same procedure as for the synthesis of PEG‐*b*‐PHPMA and PEG‐*b*‐P(HPMA‐*co*‐DMAEMA) but with the addition of protein to the pre‐polymerization solution. Stock solutions of BSA (1 mg mL^−1^), GOx (5 mg mL^−1^), and OVA (25 mg mL^−1^) in 100 mM HEPES (pH 7.3) were directly employed to synthesize protein‐loaded PSomes.

### Fluorophore Labeling of Proteins

To label proteins, a 5 mg mL^−1^ protein solution was prepared in 100 mM sodium carbonate buffer solution at pH 9.0. A 10 mM solution of the dye (RhB‐ITC or AF647‐NHS) in DMSO was directly added to the protein at a dye:protein molar ratio of 5:1. The mixture was stirred at 500 rpm at room temperature for 4 h. The labeled protein was purified using sequential PD MiniTrap G‐25 (VWR, UK) and PD MidiTrap G‐25 (VWR, UK) columns (eluent: PBS) to yield high purity labeled proteins.

### Synthesis of AlexaFluor488 Labeled PSomes

To label the PSomes, they were first synthesized with a minor incorporation of azide‐containing monomer, followed by a post‐polymerization strain promoted alkyne‐azide cycloaddition (SPAAC) reaction with dibenzocyclooctyne (DBCO)‐conjugated AF488. To synthesize azide functionalized PSomes, PEG_113_‐CDTPA (11.38 µL of 10 mg mL^−1^ stock solution in methanol, 0.02 µmol) and AzHPMA (3.9 µL of 1 mg mL^−1^ stock solution in acetone, 0.02 µmol) was added to a low protein binding microcentrifuge tube and the solvent was allowed to evaporate from the uncapped tube at room temperature until fully dried. For inert PSome synthesis: HPMA (1.14 µL, 8.43 µmol) and 8.92 µL of 100 mM HEPES buffer solution at pH 7.3 were added. For the formation of pH‐responsive PSomes: HPMA (1.03 µL, 7.59 µmol), DMAEMA (1.42 µL of 93.3 mg mL^−1^ stock solution in 100 mM HEPES buffer, 0.84 µmol) and 7.57 µL of 100 mM HEPES buffer solution at pH 7.3. The photopolymerization and purification were conducted as described above.

For fluorescent dye labeling, a solution of purified azide functionalized PSomes was prepared in DPBS at 2 mg mL^−1^, followed by the addition of DBCO‐AF488 in DMSO (10 mM) at a volume ratio of 49:1. The solution was then incubated at 37 °C for 3 h in the dark with agitation at 500 rpm. The labeled PSomes were purified with three cycles of centrifugation at 14 000 × g for 10 min.

### Encapsulation Efficiency

The encapsulation efficiency of proteins loaded in the PSomes was calculated by using a modified Micro BCA assay kit. Ice‐cold acetone was added to protein‐loaded PSomes after purification, and the solution was left at −80 °C for 30 min. The precipitated protein was separated from the polymer‐containing supernatant by centrifugation at 16 000′ g for 10 min at 4 °C. The cold acetone precipitation and the centrifugation cycle were then repeated to remove any residual polymer. The precipitated protein was resuspended in 200 µL of PBS and incubated with premixed Micro BCA reagents in a 96‐well plate for 2 h at 37 °C. After incubation, the absorbance at 562 nm was measured using a microplate reader (SpectraMax M5, Molecular Devices, San Jose, CA). Quantification was determined using BSA as a protein standard after treatment with the same precipitation process in cold acetone.

### Protease Stability of GOx and GOx‐Loaded PSomes

GOx activity was determined using a horseradish peroxidase (HRP)‐mediated 3,3′,5,5′‐tetramethylbenzidine (TMB) assay. The TMB assay buffer was prepared by mixing 8.0 mM D‐glucose and 2 µM TMB in 100 mM acetic acid/sodium acetate buffer at pH 5.5. Free GOx solution, GOx loaded PSomes and a mixture of GOx and empty PSomes (all adjusted to 100 µg mL^−1^ GOx) were incubated with 2 mg mL^−1^ proteinase K (New England Biolabs) in 100 mM Tris‐HCl pH 8.0 buffer using a thermomixer (37 °C, 500 rpm) for 7 days to assess the protection capability of PSomes by shielding the protein cargo from proteolytic enzymes. A control of free GOx solution but without proteinase K was carried out with the same conditions. After incubation, proteinase K activity was then inhibited by 1X Halt Protease Inhibitor Cocktail. To determine the remaining GOx activity, 10 µL of samples were added to a low protein binding microcentrifuge tube, followed by 10 µL of 1 µg mL^−1^ HRP and 80 µL 100 mM acetic acid/sodium acetate buffer at pH 5.5. 100 µL of the premixed TMB assay buffer was then added to the tube. The colorimetric reaction was allowed to proceed in a thermomixer (25 °C, 500 rpm) for 10 min, followed by centrifugation at 14 000 × g for 10 min to separate the supernatant. The supernatant was transferred to a 96‐well plate, and the absorbance at 650 nm was measured by UV‐Vis spectroscopy (SpectraMax M5, Molecular Devices, San Jose, CA). The activity of the control free GOx solution was set to 100% and other data was normalized to it.

### Cell Culture

MCF‐7 cells were cultured in DMEM medium with 4.5 g L^−1^ glucose and GlutaMAX supplemented with 10% (v/v) FBS and 1% (v/v) Penicillin‐Streptomycin. All cell lines were maintained at 37 °C in a cell culture incubator with 5% CO_2_ and 95% relative humidity.

BMDCs were differentiated by culturing bone marrow cells in the presence of granulocyte‐macrophage colony‐stimulating factor (GM‐CSF). In brief, bone marrow was flushed from femurs and tibias of C57Bl/6 mice using complete RPMI 1640 containing 2 mM L‐glutamine, 10% (v/v) FBS, 1% (v/v) Penicillin‐Streptomycin and 50 µM β‐ME. The cell solution was centrifuged at 400 g for 5 min and the pellet was then resuspended in 1 mL ACK lysis buffer for 5 min to remove red blood cells before dilution and resuspension in complete RPMI media. Bone marrow cells were cultured with GM‐CSF at 40 ng mL^−1^ for 6 days. Half of the media volume was replaced with fresh media containing additional growth factor at day 3 and day 6. BMDCs were then harvested by scraping in cold PBS containing 0.5 mM EDTA to detach the cells for seeding and further usage.

### In Vitro Cell Viability Assay

The 3‐(4,5‐dimethylthiazol‐2‐yl)‐5‐(3‐carboxymethoxyphenyl)‐2‐(4‐sulfophenyl)‐2H‐tetrazolium (MTS) based assay was performed to evaluate cell viability. The MTS reagent was prepared by mixing complete DMEM with CellTiter 96 AQueous One Solution reagent (Promega, USA) at a volume ratio of 5:1. The cells were seeded in a 96‐well plate at a cell density of 5 × 10^3^ cells per well and incubated for 24 h. The cells were treated with PSomes at different polymer concentrations for 24 h. The cells were then washed with PBS three times, followed by the addition of 100 µL of the working MTS reagent into each well. The plate was incubated at 37 °C for 1 h, and the absorbance at 490 nm and 630 nm was measured by UV‐Vis spectroscopy. A reference wavelength of 630 nm was used to minimize background effects from cell debris or other nonspecific sources.

### Cellular Uptake of PSomes

For flow cytometry analysis, MCF‐7 cells were seeded on a 24‐well plate at 2 × 10^5^ cells per well and incubated for 24 h. The cells were then treated with 1 mg mL^−1^ dual‐labeled PSomes for 24 h. After incubation, the cells were washed three times with PBS and detached using TrypLE Express Enzyme for 5 min. The cell solution was centrifuged at 300 rpm for 5 min, and the cells subsequently fixed with 4% (w/v) PFA in PBS at room temperature for 15 min. The fixed cells were washed twice with PBS and resuspended in PBS followed by filtration through a 40 µm mesh prior to flow cytometry to remove cell aggregates.

For confocal laser scanning microscopy, MCF‐7 cells were seeded on ibidi µ‐Slide 8 well glass bottom chambered coverslips at 5 × 10^4^ cells per well and incubated at 37 °C for 24 h. The cells were then treated with dual‐labeled PSomes at 1 mg mL^−1^ polymer concentration for 24 h. Following the incubation, the cells underwent a triple wash with PBS and were subsequently fixed with 4% (w/v) PFA in PBS at room temperature for 15 min. After fixation, the cells were counter stained with Hoechst 33342 for 10 min. The visual quantitation was validated by Mander's coefficient calculation performed on the CLSM images using the JaCoP plugin in ImageJ.^[^ ^36^ ^]^

To study the endocytosis mechanism, MCF‐7 cells were treated with CPZ (10 µg mL^−1^), EIPA (15 µg mL^−1^) and Filipin (10 µg mL^−1^) for 2 h prior to PSome treatment. After incubation with endocytosis inhibitors, the cells were treated with BSA‐AF647‐loaded PSomes for 4 h, after which the culture medium was removed, and cells were washed twice with PBS and detached with TrypLE Express Enzyme. The cells were then fixed with 4% (w/v) PFA for 15 min, followed by an analysis with a BD LSRFortessa Cell Analyzer (BD Biosciences).

### Immunofluorescent Labeling and CLSM

MCF‐7 cells were seeded on ibidi µ‐Slide 8 well glass bottom chambered coverslips at 5 × 10^4^ cells per well and incubated at 37 °C for 24 h. The media was replaced with BSA‐AF647‐loaded PSomes at 1 mg mL^−1^ in complete DMEM media, and the cells were incubated for 4 h. After incubation, the cells were washed three times with PBS and fixed with 4% (w/v) PFA in PBS at room temperature for 15 min. The cells were then permeabilized with 0.1% Triton‐X in PBS for 15 min, following by blocking with 10% (v/v) goat serum (Vector Laboratories) in PBS for 1 h at room temperature. The cells were incubated with primary antibody of EEA1 (ab70521, Abcam) and LAMP1 (ab24170, Abcam) which was diluted 1:500 in fresh PBS containing 0.1% (w/v) BSA for 1 h. Secondary antibody staining with goat anti‐mouse IgG Alexa‐Fluor 555 for EEA and goat anti‐rabbit IgG Alexa‐Fluor 555 for LAMP1 (diluted 1:500 in fresh PBS containing 0.1% w/v BSA) was performed at room temperature for 1 h. The nuclei of the cells were finally stained by treatment with Hoechst 33342 for 10 min. Imaging was performed using a confocal microscope (Leica SP8 inverted confocal microscope, Leica Microsystems, Germany) and the images were processed using Image J software.

### Immunocytochemistry for STORM

MCF‐7 cells were seeded on ibidi µ‐Slide 8 well glass bottom chambered coverslips at 5 × 10^4^ cells per well and incubated at 37 °C for 24 h. For bafilomycin A1 pretreatment groups, cells were pre‐treated with complete media containing 100 nM bafilomycin A1 (Cell Signaling Technology) prior to incubation with PSomes for 1 h. Cells were treated with PSome/AF647‐BSA at 1 mg mL^−1^ polymer concentration for 4 h. After incubation, the cells were washed three times with pre‐warmed PBS and then fixed with PBS containing 4% (w/v) PFA and 0.2% (w/v) glutaraldehyde at room temperature for 15 min. After fixation, samples were stored at 4 °C before staining and imaging. Immunostaining was performed immediately prior to STORM image acquisition. Cells were permeabilized with 0.05% (v/v) Triton X‐100 in PBS for 5 min at RT and blocked with 3% (w/v) BSA in PBS for 2 h at RT. For primary antibody staining, cells were treated with LAMP1 antibody (D2D11, Rabbit mAb, Cell Signaling Technology) (diluted 1:200 in the blocking solution), incubated at 4 °C overnight, followed by 90 min incubation with the secondary antibody (donkey anti‐rabbit IgG Alexa‐Fluor 488 Invitrogen or donkey anti‐rabbit IgG Alexa‐Fluor 647 Invitrogen) (diluted 1:1000 in the blocking solution). Finally, the antibodies were fixed with 2% (w/v) PFA for 10 min at room temperature, and nuclei were stained with 4′,6‐diamidino‐2‐phenylindole (DAPI; Invitrogen, D1306; 1:5000 dilution in PBS) for 5 min at room temperature. For STORM imaging, an imaging buffer with the following composition was added to the well: Tris buffer (160 mM Tris, 40 mM NaCl, pH 8.0), 10% (w/v) glucose, 0.5 mg mL^−1^ glucose oxidase, 47 µg mL^−1^ catalase, and 10 mM MEA (cystamine, pH 8.0).

### In Vitro Intracellular HRP Activity

The intracellular HRP activity was determined by using a TMB assay after incubation of MCF‐7 cells with HRP‐loaded pH‐responsive PSomes. To assess this, MCF‐7 cells were seeded in a 96‐well plate at a cell density of 5 × 10^3^ cells per well and incubated for 24 h. The cells were then treated with free HRP, empty pH‐responsive PSomes, and HRP‐loaded pH‐responsive PSomes (all adjusted to 100 ng mL^−1^ HRP) for 24 h. After incubation, the cells were washed three times with PBS and 100 µL of the premixed TMB assay buffer was then added to each well. Enzyme activity kinetics were then measured by UV‐Vis spectroscopy (SpectraMax M5, Molecular Devices, San Jose, CA) at a wavelength of 650 nm for 15 min with 30 s intervals between measurements.

### In Vitro Dendritic Cell Activation

To assess dendritic cell maturation and antigen cross‐presentation in vitro, BMDCs were seeded on a 24‐well plate at 2 × 10^5^ cells per well and incubated for 24 h. The cells were treated with 1 mL of free OVA (20 µg mL^−1^ of OVA), OVA‐loaded inert PSomes, and OVA‐loaded pH‐responsive PSomes (adjusted to 20 µg mL^−1^ OVA) for 24 h. Control experiments were conducted by incubation with empty inert PSomes and pH‐responsive PSomes (adjusted to the same polymer concentration as OVA‐loaded PSomes). After incubation, BMDCs were harvested, and resuspended in PBS containing 1% (w/v) BSA. The cells were incubated with anti‐CD16/32 at room temperature for 15 min to block non‐specific binding of immunoglobulin to Fc receptors, followed by a 30 min incubation with fluorophore‐labeled antibodies against CD11c (BioLegend, 1:200 dilution in PBS containing 1% (w/v) BSA), CD86 (BD Bioscience, 1:500 dilution in PBS containing 1% (w/v) BSA), CD80 (BD Bioscience, 1:500 dilution in PBS containing 1% (w/v) BSA) and SIINFEKL‐H2K^b^ (BioLegend, 1:500 dilution in PBS containing 1% (w/v) BSA) prior to analysis by flow cytometry. The data were analyzed using FlowJo software.

### Statistical Analyses

Data was processed using GraphPad Prism 10. Where applicable, data has been represented as mean ± standard deviation with at least three replicates for each analysis. Detailed information on data pre‐processing, sample sizes, and statistical methods, including post‐hoc tests, are described further within the figure captions.

## Conflict of Interest

M.M.S. invested in, consults for (or was on scientific advisory boards or boards of directors) and conducts sponsored research funded by companies related to the biomaterials field. The rest of the authors declare no competing financial interest.

## Author Contributions

C.T. and O.R.‐G. contributed equally to this work. C.T., O.R.‐G., and J.Y. conceived the project concept and designed the experiments. C.T. prepared and characterized the polymersomes and performed all in vitro experiments. M.O. performed the STORM experiments and analysis. A.N. performed the FCS measurements and analysis. H.K. assisted with flow cytometry, confocal microscopy, and analysis. S.E.B. and M.C. assisted with Cryo‐TEM and analysis. D.J.P and C.L. prepared the bone marrow‐derived dendritic cells. C.T., O.R.‐G., and J.Y. wrote the manuscript with feedback from all authors. M.M.S. and J.Y. supervised the project.

## Supporting information



Supporting Information

## Data Availability

The data that support the findings of this study are available from the corresponding author upon reasonable request.
