# Supplementary material for: Unlocking Intracellular Protein Delivery by Harnessing Polymersomes Synthesized at Microliter Volumes using Photo‐PISA
Source: Adv Mater. 2024 Oct 17;36(49):2408000. doi: 10.1002/adma.202408000 (PMC11619233; doi:10.1002/adma.202408000)
Supplement: Supplementary file 1 — Supporting Information [file ADMA-36-2408000-s001.docx]

**Supplementary Information**

**Unlocking intracellular protein delivery by harnessing polymersomes synthesized at microliter volumes using Photo-PISA**

Chalaisorn Thanapongpibul,^1†^ Omar Rifaie-Graham,^1†^ Miina Ojansivu,^2^ Adrian Najer,^1^ Hyemin Kim,^1^ Saskia E. Bakker,^3^ Mohamed Chami,^4^ David J. Peeler,^1^ Chenchen Liu,^1,5^ Jonathan Yeow,^1,6^* Molly M. Stevens^1,2,5^*

^1^Department of Materials, Department of Bioengineering, and Institute of Biomedical Engineering, Imperial College London, London, SW7 2AZ, UK

^2^Department of Medical Biochemistry and Biophysics, Karolinska Institutet, Stockholm, 17177, Sweden

^3^Advanced Bioimaging Research Technology Platform, University of Warwick, Gibbet Hill Road, Coventry, CV4 7AL, UK

^4^BioEM Lab, Biozentrum, University of Basel, Basel, 4058, Switzerland

^5^Kavli Institute for Nanoscience Discovery, Department of Physiology, Anatomy and Genetics, Department of Engineering Science, and Kavli Institute for Nanoscience Discovery, University of Oxford, Oxford, OX1 3QU, UK

^6^Graduate School of Biomedical Engineering, University of New South Wales, Sydney, NSW, 2052, Australia

Corresponding authors: [j.yeow@unsw.edu.au](mailto:j.yeow@unsw.edu.au), [molly.stevens@dpag.ox.ac.uk](mailto:molly.stevens@dpag.ox.ac.uk)

^†^ Authors contributed equally

**Supplementary Information**

**Section S1. DLS analysis of PEG-*b*-PHPMA and PEG-*b*-P(HPMA-*co*-DMAEMA) PSomes**

DLS measurements were employed to evaluate the hydrodynamic diameter (D_H_) of PEG-*b*-PHPMA and PEG-*b*-P(HPMA-*co*-DMAEMA) PSomes. When transitioning from pH 7.4 (PBS) to pH 5.5, an increase in the polydispersity indexes (PDIs) for PEG-*b*-P(HPMA-*co*-DMAEMA) PSomes was observed, whilst remaining fairly constant for PEG-*b*-PHPMA PSomes (Figure S4A). However, the intensity-derived measurements revealed that the calculated D_H_ remained consistent for both types of PSomes (Figure S4B). This lack of change could be attributed to the fact that intensity measurements in DLS are dominated by the scattering of larger particles which therefore contribute more substantially to the overall intensity-based distribution.^[1]^ Therefore, it was necessary to refer to the number-based distribution of the PSomes under varying pH conditions (with support from DLS derived count rates), as this enables a more realistic representation of the size of the particle population, particularly in the case of smaller (< 20 nm) nanoparticles (Figure 2F,G).

**Section S2. Proteolysis resistance of loaded protein within PSomes**

To further verify that the BSA associated to the PSomes was encapsulated rather than physically absorbed, we investigated the ability of the PSome membrane to protect the protein cargo from external degrading agents such as proteases. To assess this, we encapsulated glucose oxidase (GOx) as a model protein with enzymatic activity that can be readily monitored with a colorimetric assay and proteinase K as a broad-spectrum protease. When free GOx (100 µg mL^-1^) was treated with proteinase K (2 mg mL^-1^), it lost most of its enzymatic activity. In contrast, GOx loaded within either inert or pH-responsive PSomes (adjusted to 100 µg mL^-1^ of encapsulated GOx based on the Micro BCA assay) was highly resistant to digestion by proteinase K and retained comparable GOx activity to free GOx solution (without proteinase K) even after 7-days of incubation (Figure S7). Additionally, control experiments whereby empty PSome dispersions were spiked with exogenous GOx and then incubated with proteinase K displayed negligible GOx activity.

**Section S3. The cellular internalization mechanism of protein-loaded PSomes**

One factor influencing cellular uptake, particularly in its susceptibility to inhibition, is the impact of cold temperatures. Decreased temperatures, specifically at 4 °C, can inhibit energy-dependent uptake mechanisms into cells and therefore can be used to probe whether uptake of particles into cells is occurring in an energy-dependent manner.^[2]^ To investigate this effect, cells were incubated with AF647-BSA-loaded inert and pH-responsive PSomes at 4 °C, which resulted in significantly reduced uptake in comparison to cells incubated at 37 °C (Figure S11). To further investigate the mechanism of internalization, the cellular uptake of BSA-AF647 loaded PSomes was investigated in MCF-7 cells which had been pre-treated with a variety of endocytosis inhibitors. Under these conditions, only 5-(*N*-ethyl-*N*-isopropyl) amiloride (EIPA) and chlorpromazine (CPZ), which inhibit macropinocytosis and clathrin-mediated endocytosis, respectively, caused a significant reduction in the uptake of BSA-AF647 loaded inert and pH-responsive PSomes (Figure S11).

**Supplementary Figures**


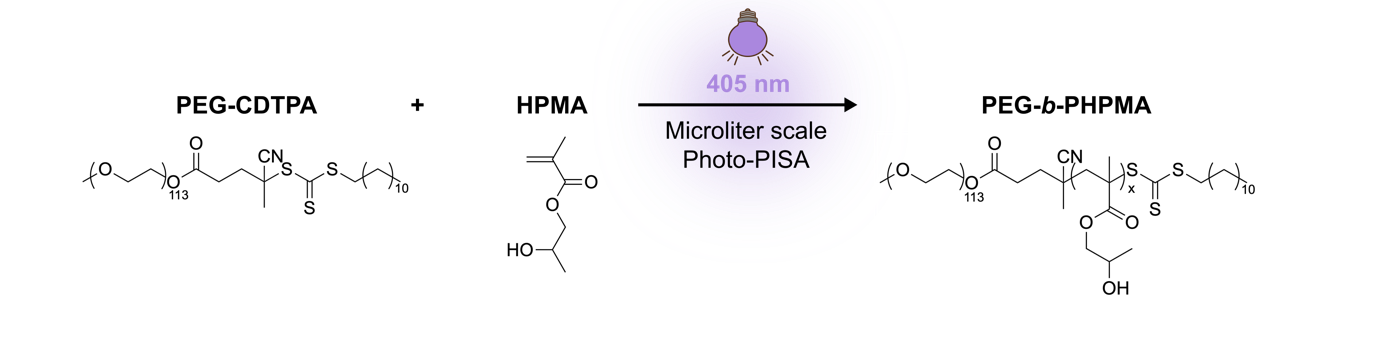


**Figure S1.** Scheme of PEG-*b*-PHPMA synthesis via photoinitiated PISA at a 10 μL scale.


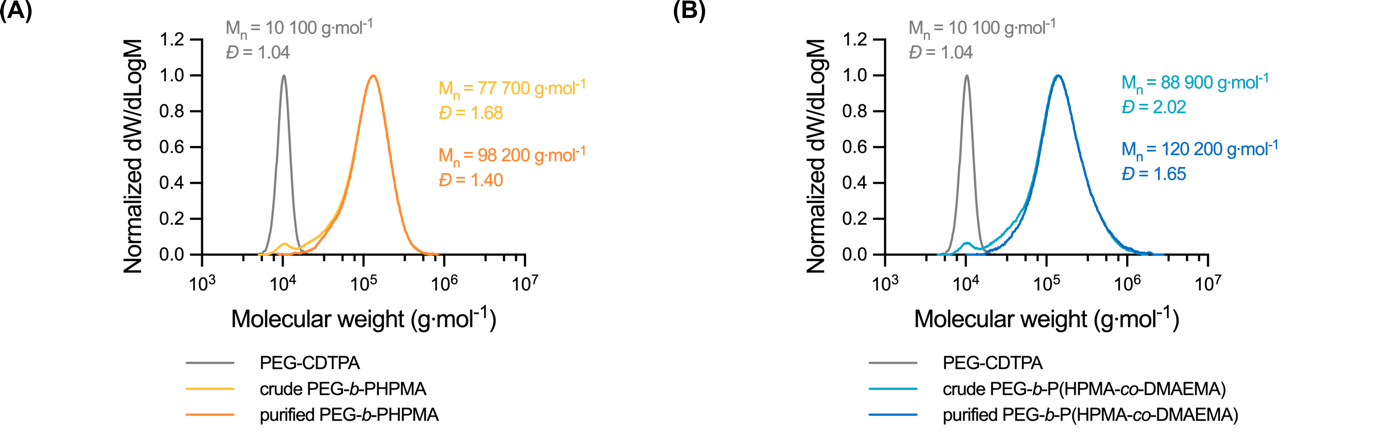


**Figure S2.** Molecular weight distribution of crude and purified (A) PEG-*b*-PHPMA and (B) PEG-*b*-P(HPMA-*co*-DMAEMA) block copolymers compared to PEG-CDTPA. Polymers were purified by three cycles of centrifugation.


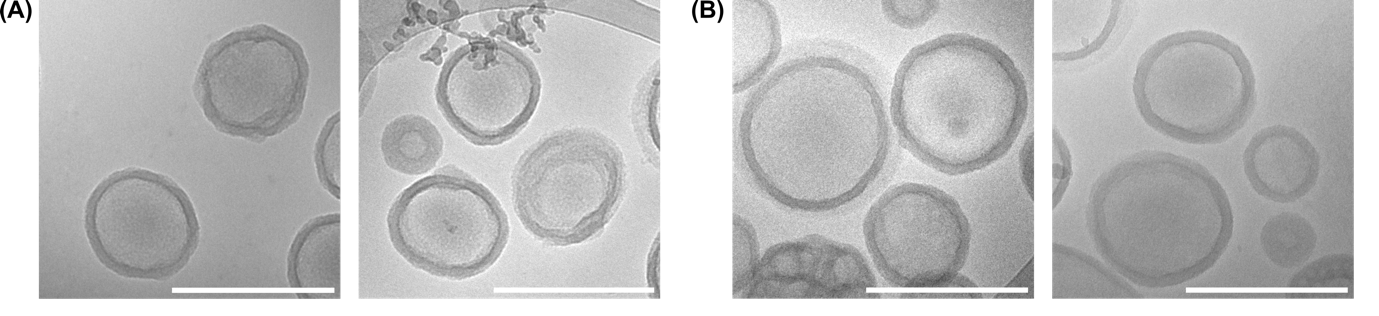


**Figure S3**. Additional cryo-TEM images of (A) PEG-*b*-PHPMA and (B) PEG-*b*-P(HPMA-*co*-DMAEMA) PSomes. Scale bars: 500 nm.


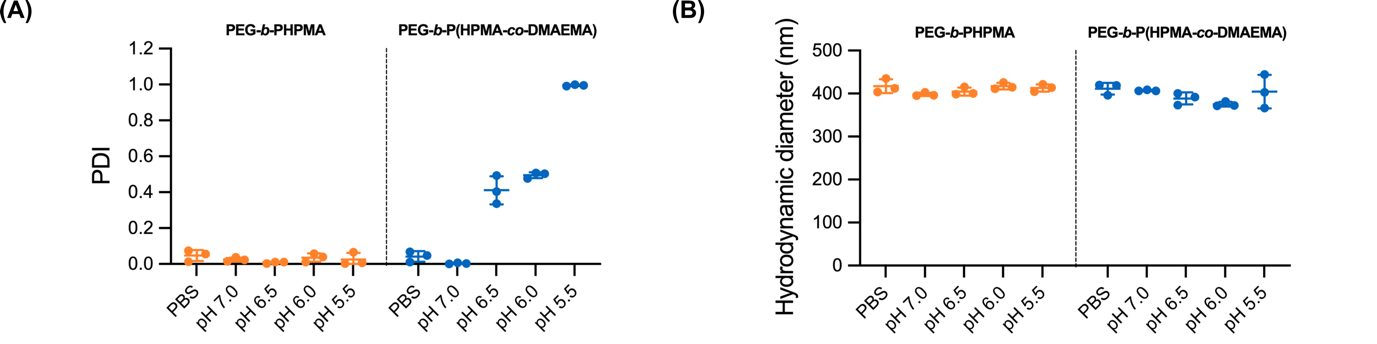


**Figure S4.** Changes in PSome populations at various pH states as determined by DLS. (A) PDI and (B) intensity-derived average hydrodynamic diameters of PEG-*b*-PHPMA PSomes (orange) and PEG-*b*-P(HPMA-*co*-DMAEMA) PSomes (blue) against pH (*n* = 3, technical replicates).


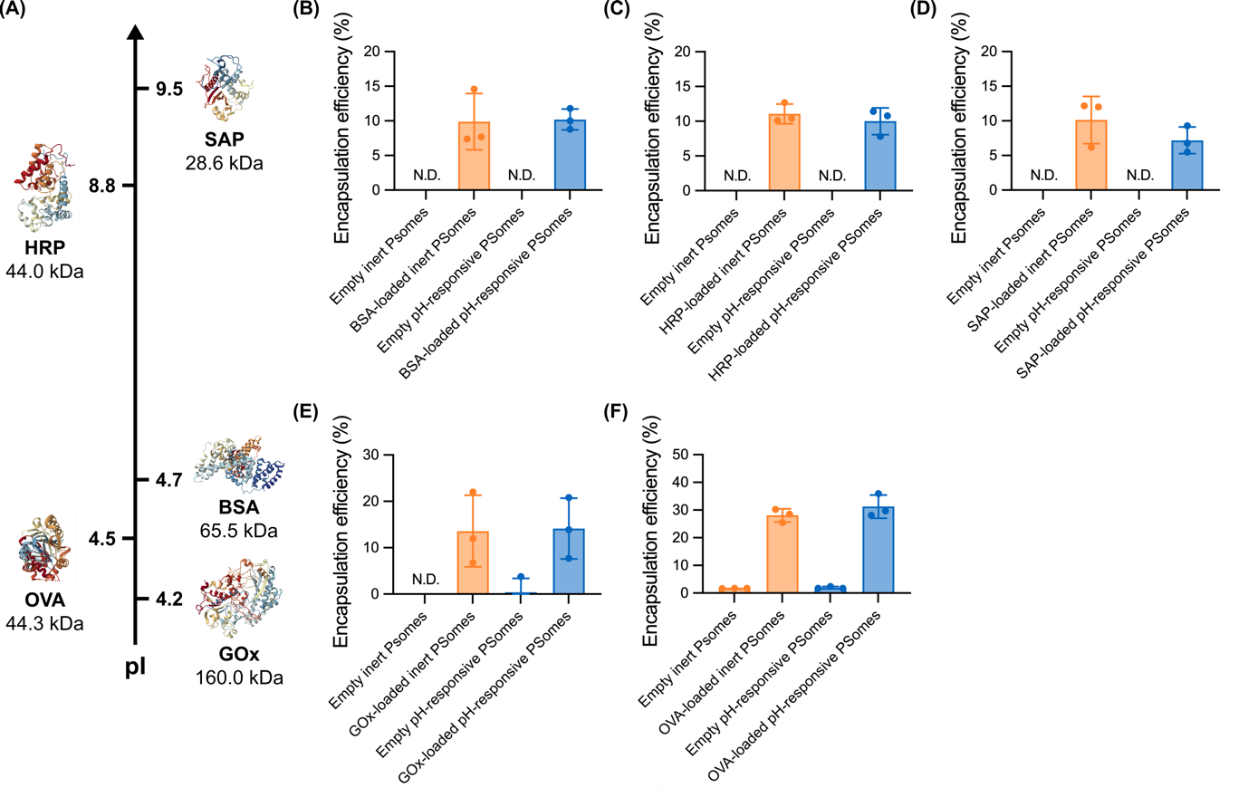


**Figure S5.** Encapsulation efficiency of protein-loaded PSomes. (A) Model proteins employed in this study with different molecular weights and isoelectric points (pI). Encapsulation efficiency (EE%) of PSomes loaded with (B) BSA (1 mg mL^-1^; EE% = 9.9 ± 4.1% for inert PSomes, 10.2 ± 1.5% for pH-responsive PSomes), (C) HRP (1 mg mL^-1^; EE% = 11.1 ± 1.4% for inert PSomes, 10.0 ± 1.9% for pH-responsive PSomes), (D) SAP (1 mg mL^-1^; EE% = 10.1 ± 3.4% for inert PSomes, 7.2 ± 1.9% for pH-responsive PSomes), (E) GOx (5 mg mL^-1^; EE% = 13.6 ± 7.7% for inert PSomes, 14.1 ± 6.6% for pH-responsive PSomes), and (F) OVA (25 mg mL^-1^; EE% = 28.1 ± 2.4% for inert PSomes, 31.3 ± 4.1% for pH-responsive PSomes). The encapsulation efficiency of each protein was calculated using a modified Micro BCA^TM^ assay whereby free polymer is removed *via* precipitation of the loaded protein in cold acetone. The initial amount of protein added for the synthesis was set to 100% (mean ± SD, *n* = 3, synthetic replicates, N.D.: not detected). The final protein concentrations of protein-loaded inert and pH-responsive PSomes were as follows: BSA at 0.10 mg mL^-1^ and 0.10 mg mL^-1^; HRP at 0.11 mg mL^-1^ and 0.10 mg mL^-1^; GOx at 0.15 mg mL^-1^ and 0.15 mg mL^-1^; SAP at 0.10 mg mL^-1^ and 0.07 mg mL^-1^; and OVA at 0.28 mg mL^-1^ and 0.31 mg mL^-1^, respectively.


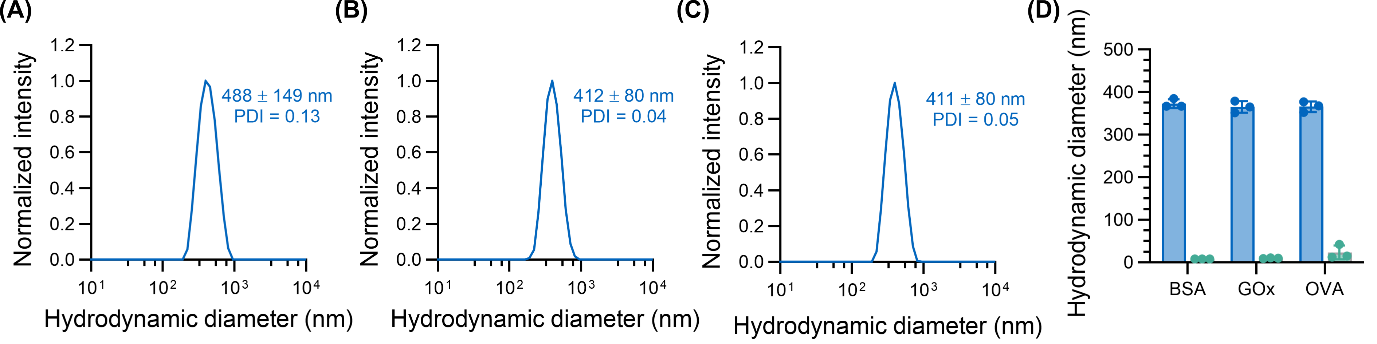


**Figure S6.** Average hydrodynamic diameters and pH-responsiveness of protein-loaded PSomes. DLS intensity-based distributions of pH-responsive PSomes loaded with (A) BSA (1 mg mL^-1^), (B) GOx (5 mg mL^-1^), and (C) OVA (25 mg mL^-1^) (*n* = 3, technical replicates). (D) Number-based average hydrodynamic diameter of different proteins loaded into pH-responsive PSomes after incubation in PBS (blue) and pH 5.5 buffer (green) (*n* = 3, technical replicates).

**
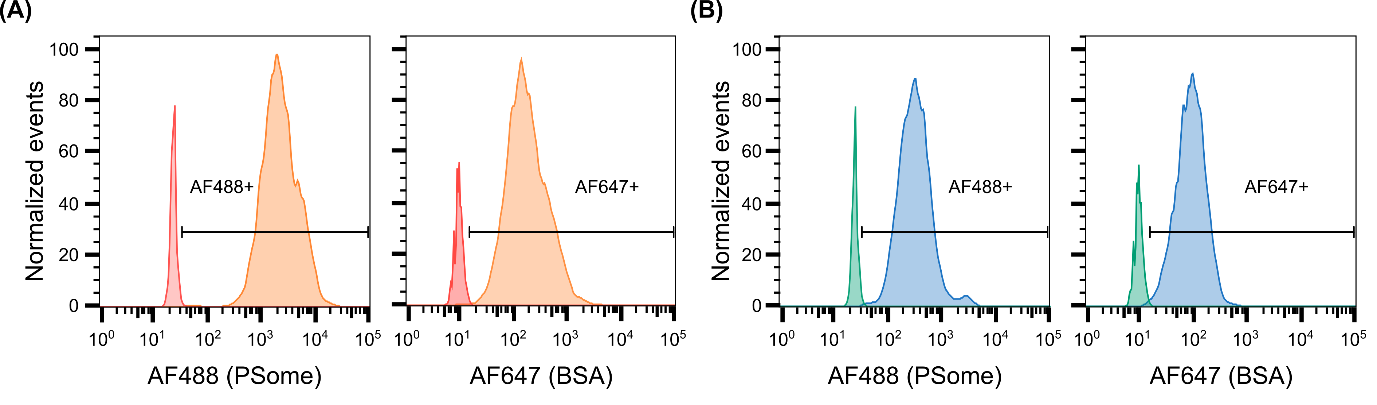
**

**Figure S7.** Characterization of protein loading into polymersomes by NanoFCM. Histograms of PSome-AF488 and BSA-AF647 for (A) inert PSomes and (B) pH-responsive PSomes with loaded BSA. Unlabeled inert PSomes (red; MFI: AF488 = 24.1, AF647 = 9.9) and pH-responsive PSomes (green; MFI: AF488 = 25.1, AF647 = 10) are represented, while dual-labeled inert PSomes (orange; MFI: AF488 = 2975, AF647 = 267) and pH-responsive PSomes (blue; MFI: AF488 = 423, AF647 = 108) are represented (*n* = 4,000-10,000 particles).


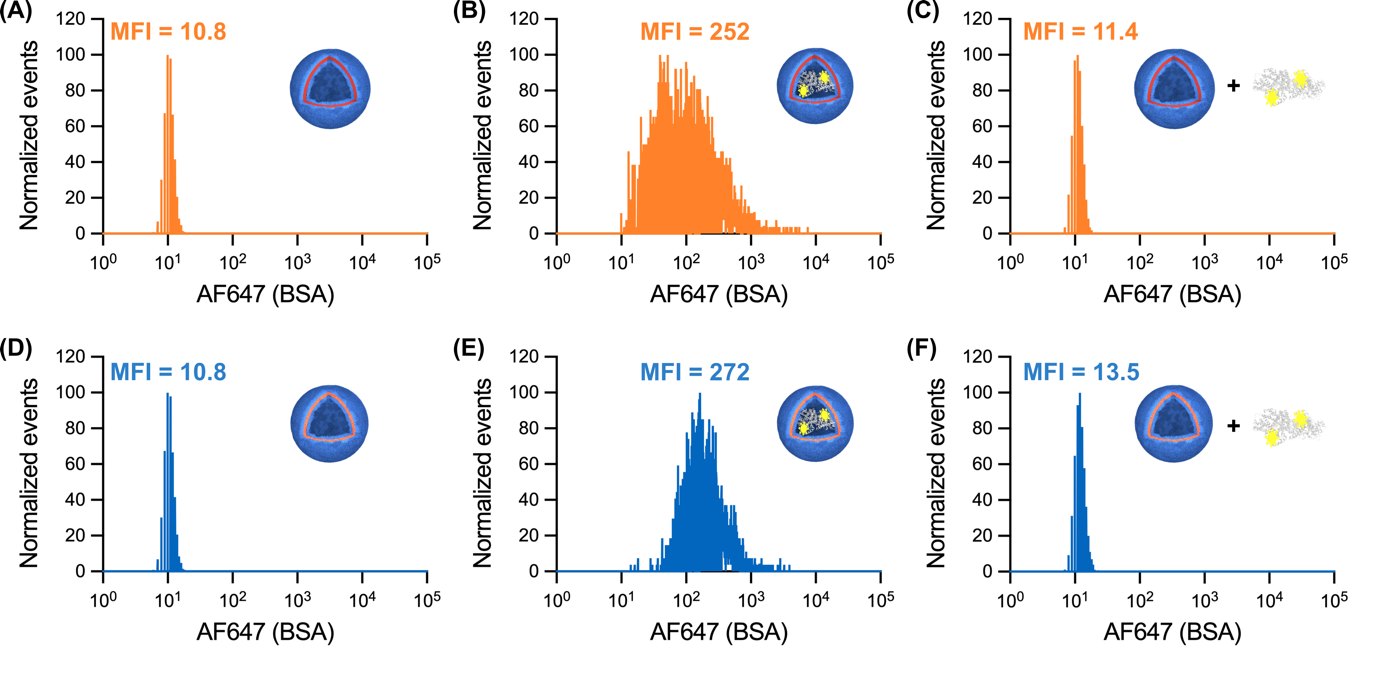
**Figure S8.** Characterization of protein loading into polymersomes by NanoFCM. Mean fluorescence intensity (MFI) and histograms of BSA-AF647 for (A) empty inert PSomes, (B) inert PSomes with loaded BSA-AF647, (C) empty inert PSomes spiked with free BSA-AF647, (D) empty pH-responsive PSomes, (E) pH-responsive PSomes with loaded BSA-AF647 and (F) empty pH-responsive PSomes spiked with free BSA-AF647 (*n* = 4,000-10,000 particles).


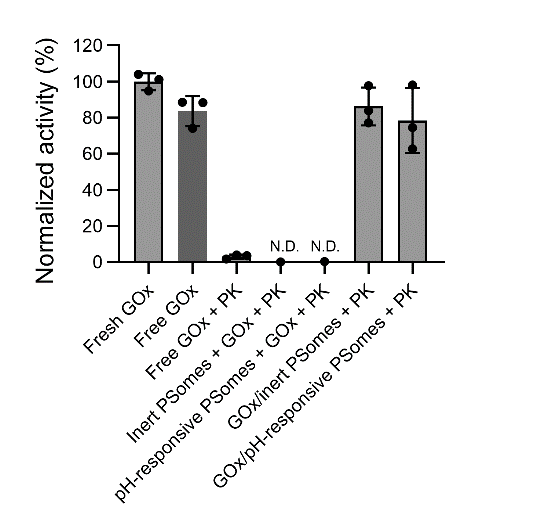


**Figure S9.** Protection of GOx-loaded inert and pH-responsive PSomes from proteolytic degradation. Free GOx after irradiation with 405 nm light for 3 h, GOx-loaded PSomes, and a physical mixture of empty PSomes with free GOx were incubated with proteinase K for 7 days at 37 °C. Then, the GOx activity was developed by an HRP/TMB assay (mean ± SD, *n* = 3, N.D.: not detected) and compared against a control of fresh GOx.


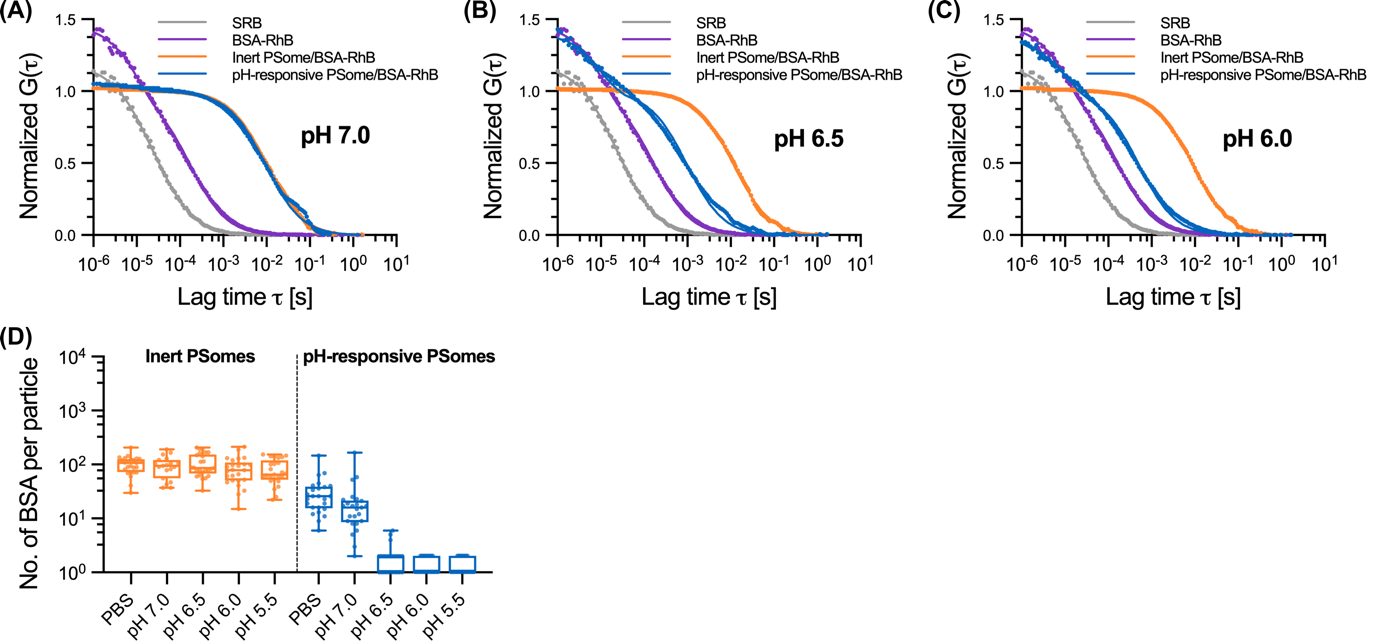


**Figure S10.** Determination of protein release at different pH conditions by FCS. Normalized FCS autocorrelation curves of sulforhodamine B (SRB), BSA-RhB, inert PSome/BSA-RhB and pH-responsive PSome/BSA-RhB in (A) pH 7.0, (B) pH 6.5 and (C) pH 6.0 buffer (average curves of *n* = 25 individual measurements, dots represent raw data, straight lines are fitted curves). (D) FCS derived number of BSA molecules per particle for inert PSomes/BSA-RhB and pH-responsive PSomes/BSA-RhB over a range of pH values *(n* = 25 individual measurements)*.* Box plots: center line, median; box limits, upper and lower quartiles; whiskers, minimum and maximum values.


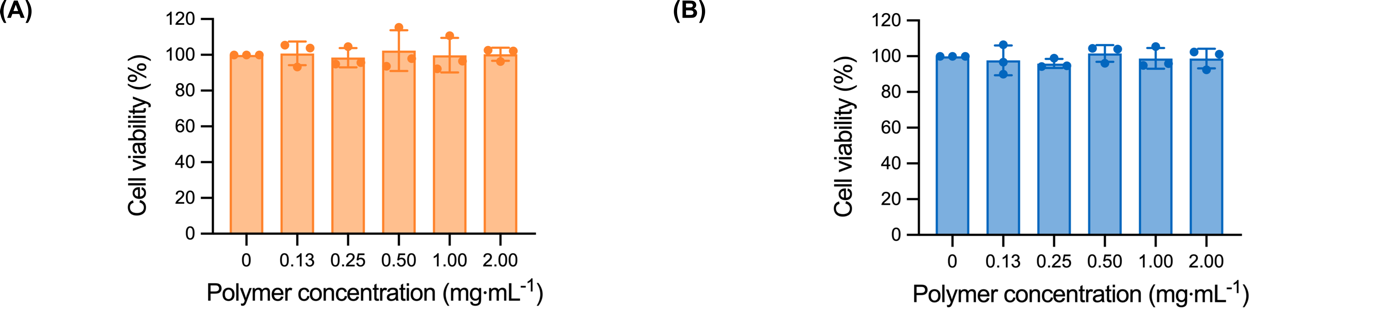


**Figure S11.** Cell viability assessed by MTS assay of MCF-7 cells. Viability determined after incubation with (A) inert PSomes and (B) pH-responsive PSomes for 24 h (mean ± SD, *N* = 3, *n* = 3).


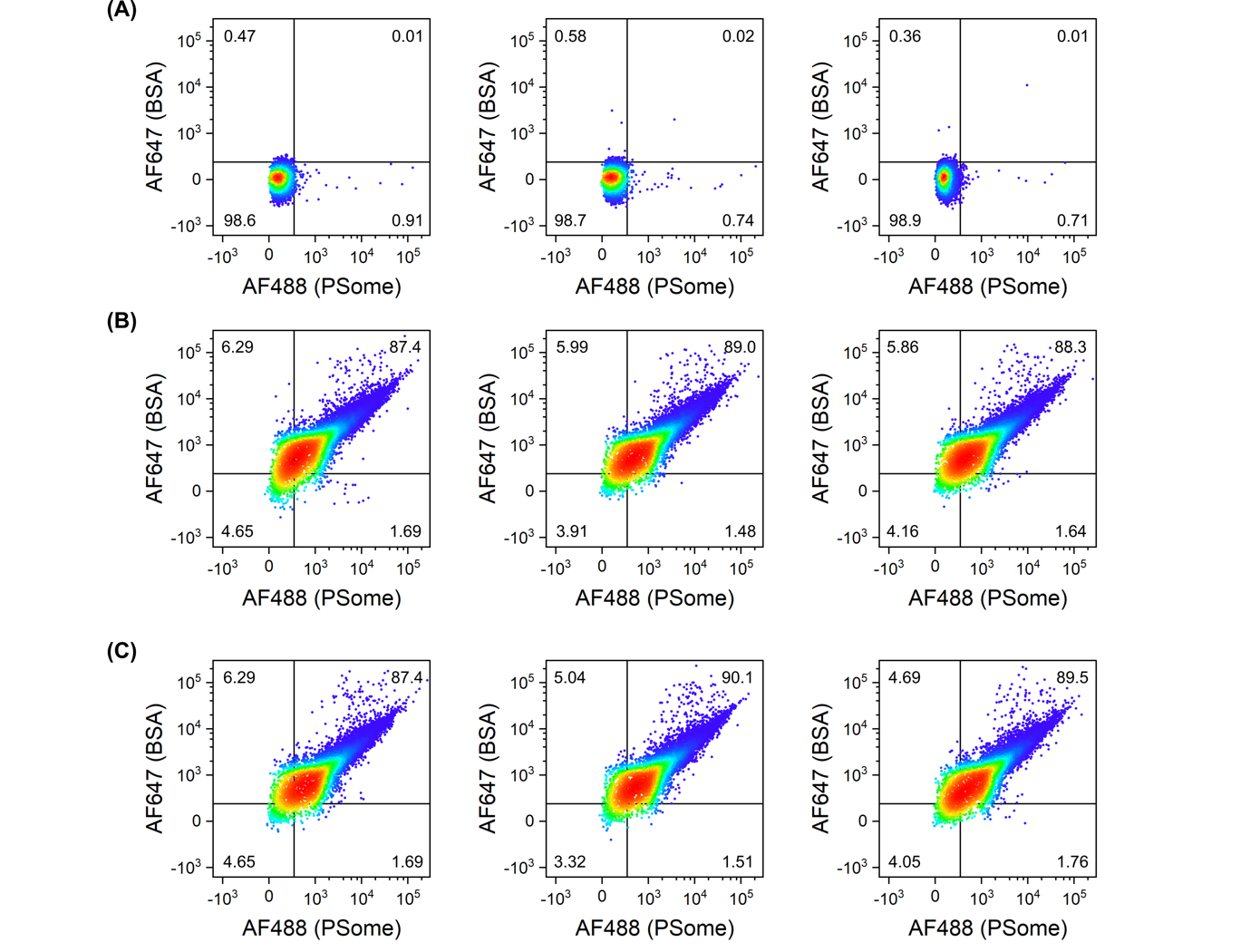


**Figure S12.** Flow cytometry analysis of MCF-7 cells after incubation with labelled PSomes. Cells were incubated with: (A) PBS (*n* = 3, technical replicates), (B) dual-labeled inert PSome-AF488/BSA-AF647 (*n* = 3, technical replicates), and (C) dual-labeled pH-responsive PSome-AF488/BSA-AF647 (*n* = 3, technical replicates) for 24 h.


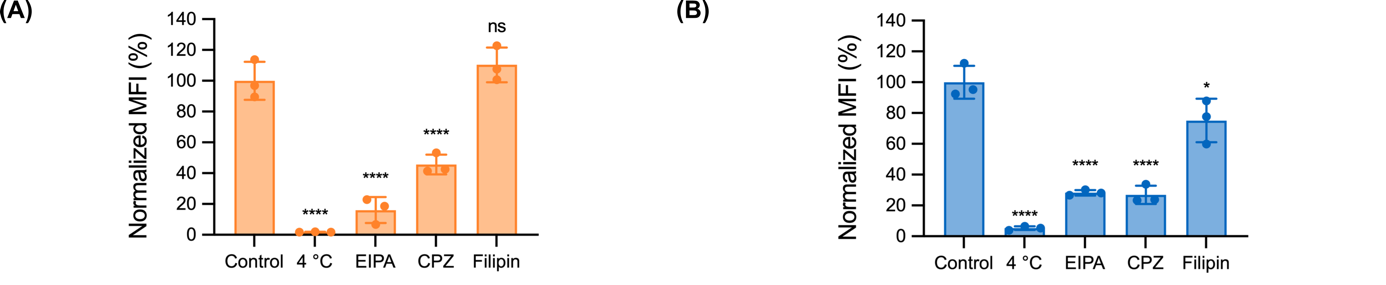


**Figure S13.** Mechanism of PSome uptake by MCF-7 cells as determined by flow cytometry. Effect of low temperatures (4 °C) and various endocytosis inhibitors (EIPA, CPZ, and Fillipin) on the uptake of (A) inert PSome/BSA-AF647 and (B) pH-responsive PSome/BSA-AF647 by MCF-7 cells, after 4 h of incubation with the PSomes (mean ± SD, *n* = 3, technical replicates). Statistical significance was determined using one-way ANOVA with Dunnett’s multiple comparisons test. *****p* < 0.0001, **p* < 0.05, *ns* not significant *versus* control (normal uptake at 37°C).

**
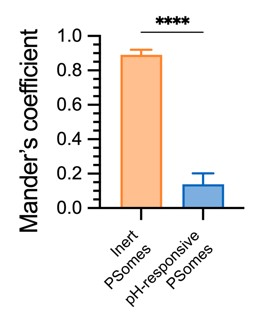
**

**Figure S14.** Quantification of colocalization between the loaded BSA and either inert or pH-responsive PSomes within MCF-7 cells after incubation with dual-labeled inert PSome-AF488/BSA-AF647 and dual-labeled pH-responsive PSome-AF488/BSA-AF647 for 24 h. Colocalization analysis was performed using the JaCoP^[3]^ plugin in ImageJ to obtain Manders’ overlap coefficients of BSA with the PSome signal. The Manders’ coefficient indicates the fraction of the total fluorescent pixels of BSA-AF647 that overlap with the fluorescent pixels associated with PSome-AF488. This coefficient ranges from 0 to 1, with values close to 1 indicating a high degree of overlap, reflecting the spatial overlap and correlation between the two fluorophores (mean ± SD, *n* = 90-100 cells). Statistical significance was determined by an unpaired Student’s t-test. *****p* < 0.0001.


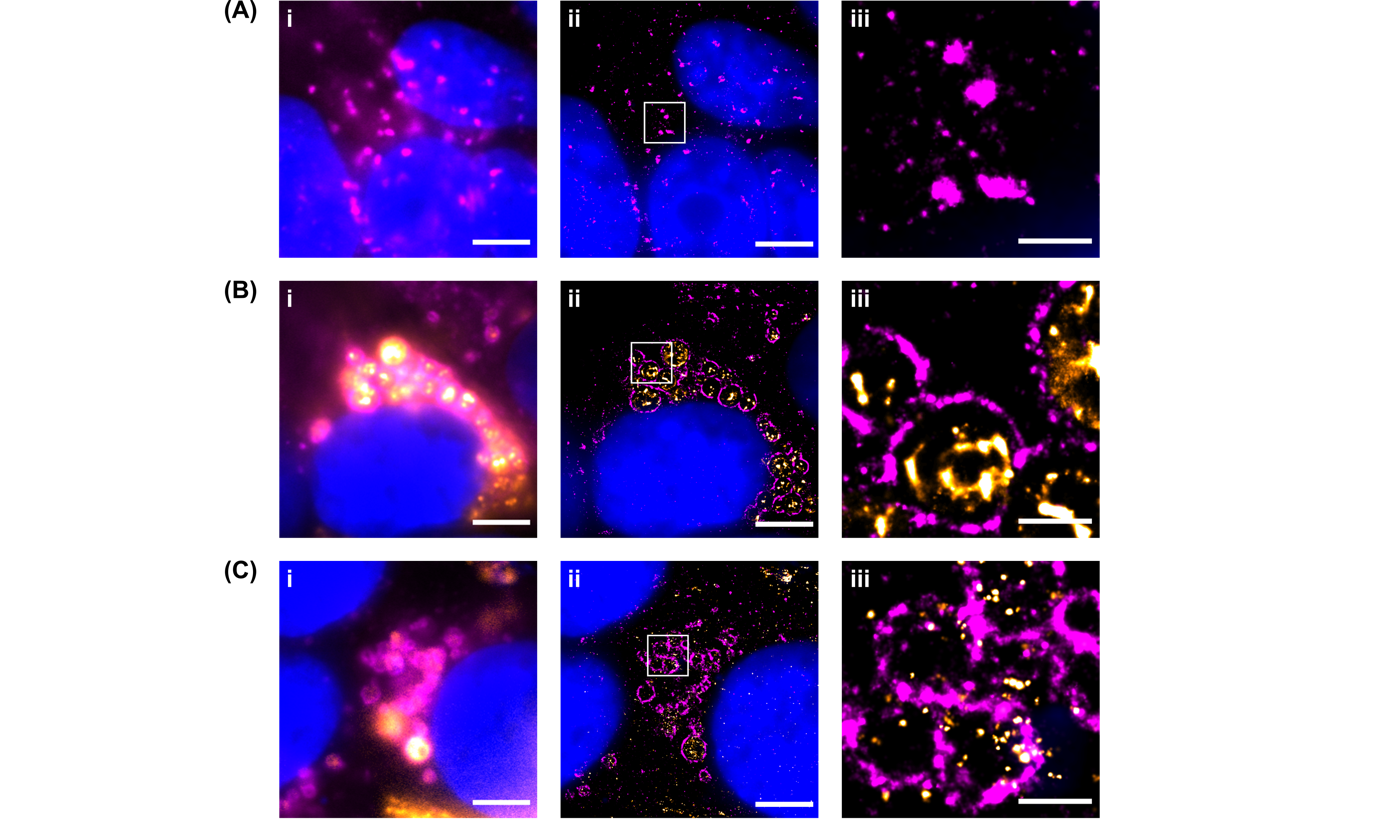


**Figure S15.** STORM visualization of MCF-7 cells after 24 h incubation with protein-loaded PSomes. Two-color super resolution STORM images of BSA-loaded PSomes and lysosomal compartments of MCF-7 cells treated with (A) PBS, (B) inert PSomes and (C) pH-responsive PSomes after 24 h of incubation. Nuclei were stained by DAPI (blue), the late endosomes were stained with AF488-labeled anti-LAMP1 (magenta), and BSA as a model protein was labelled with AF647 (yellow). For images in A – C: (i) diffraction-limited image; (ii) STORM reconstructed image; (iii) zoom-in of STORM reconstruction. Scale bars (i – ii): 5 μm. Scale bars (iii): 1 μm.

**
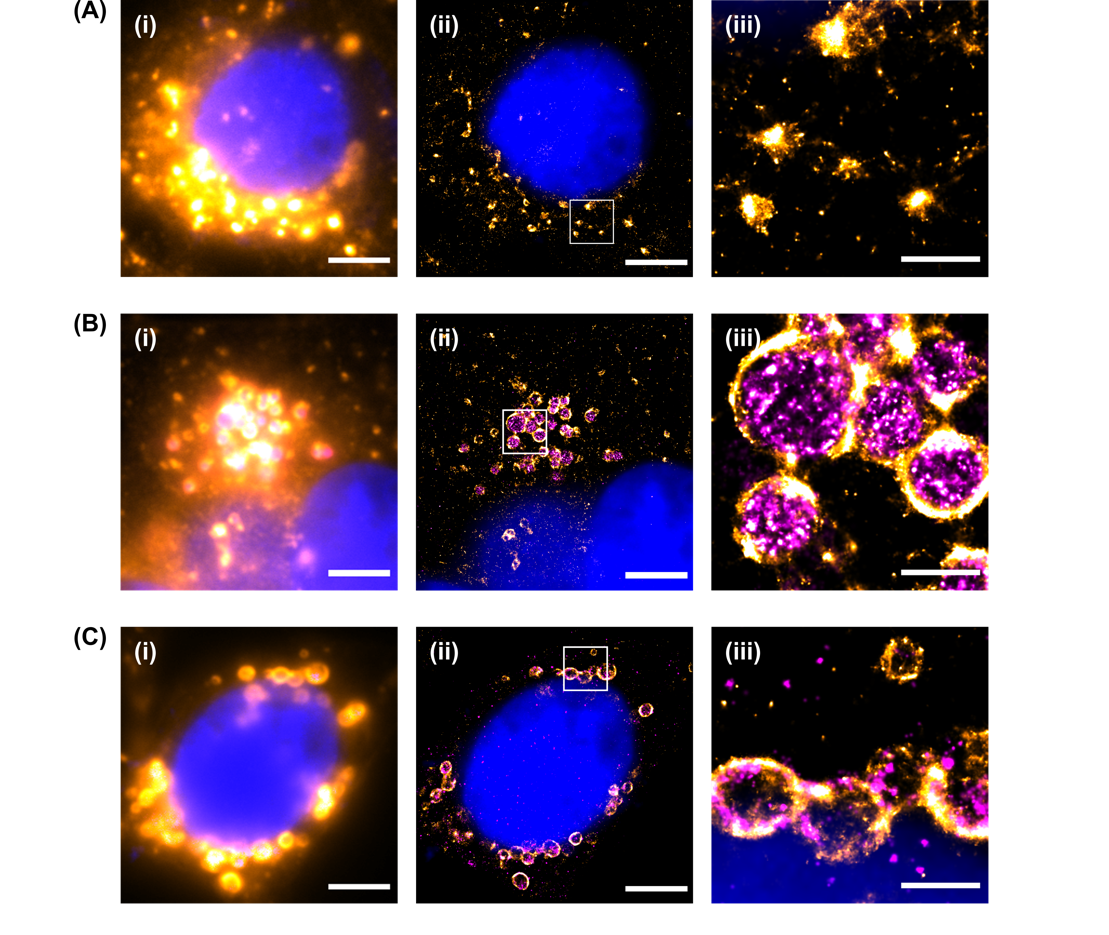
**

**Figure S16.** Representative two-color super resolution STORM images of AF488-labeled PSomes and lysosomal compartments after 24 h of incubation. MCF-7 cells incubated with (A) PBS, (B) AF488-labeled inert and (C) pH-responsive PSomes for 24 h. Nuclei were stained with DAPI (blue), the polymersome was labeled with AF488 (magenta), and the late endosomes were stained with AF647-labeled anti-LAMP1 (yellow). For images in A – C: (i) diffraction-limited image; (ii) STORM reconstructed image; (iii) zoom-in of STORM reconstruction. Scale bars (i – ii): 5 μm. Scale bars (iii): 1 μm.


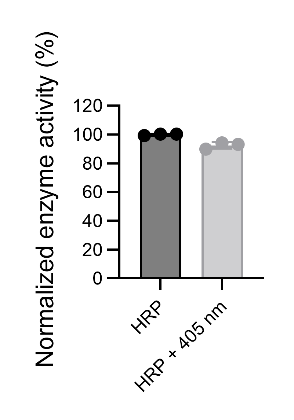


**Figure S17.** Retention of enzyme activity of HRP before and after light irradiation. Activity of HRP was measured by the TMB assay before and after irradiation with 405 nm light for 3 h. (mean ± SD, *n* = 3, technical replicates)


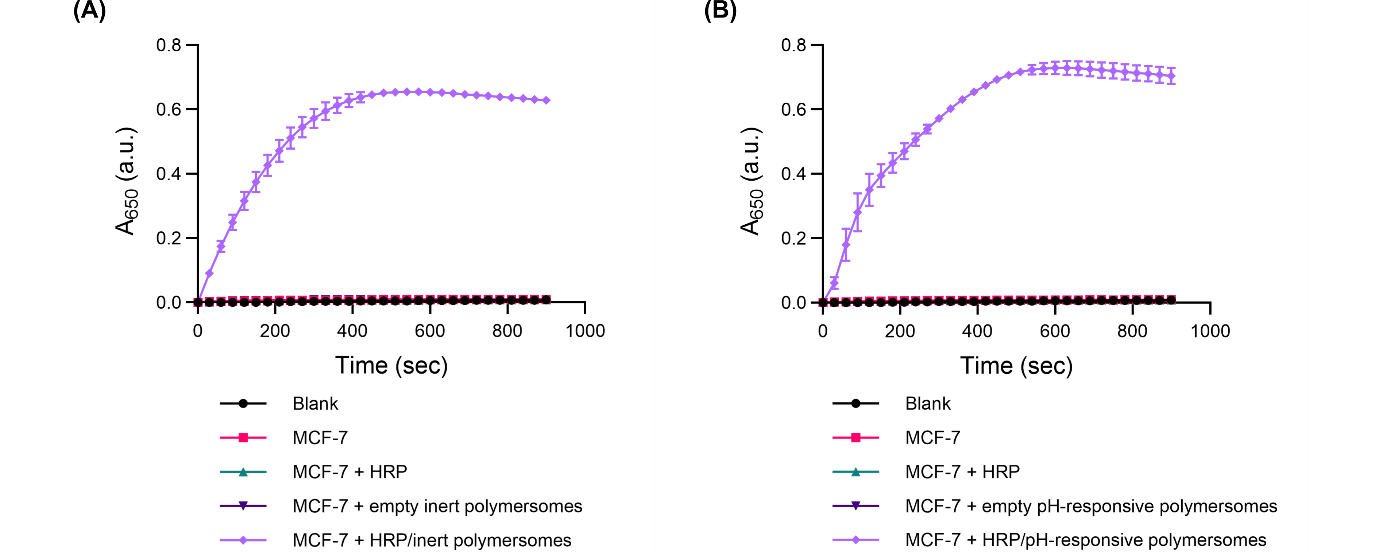


**Figure S18.** *In vitro* kinetic measurements of HRP activity as measured by UV-Vis spectroscopy at 650 nm. An *in vitro* TMB assay for peroxidase activity of the HRP after 24 h incubation of MCF-7 cells with free (unformulated) HRP, empty PSomes and HRP-loaded PSomes. For clarity, data has been plotted separately for (A) inert PSomes and (B) pH-responsive PSomes (*n* = 3, technical replicates).

**Additional references**

[1] J. Stetefeld, S. A. McKenna, T. R. Patel, *Biophys. Rev.* **2016**, 8, 409.

[2] J. J. Rennick, A. P. R. Johnston, R. G. Parton, *Nat. Nanotechnol.* **2021**, 16, 266.

[3] S. Bolte, F. P. Cordelieres, *J. Microsc.* **2006**, 224, 213.
